# USP9X-triggered ferroptosis mediates follicular atresia via deubiquitinating Beclin1 in chicken

**DOI:** 10.1186/s40104-025-01269-8

**Published:** 2025-10-23

**Authors:** Yuqi Chen, Wenjuan Wang, Can Cui, Yao Zhang, Zhuanjian Li, Huadong Yin, Shunshun Han

**Affiliations:** 1 Key Laboratory of Livestock and Poultry Multi-Omics, Ministry of Agriculture and Rural Affairs, College of Animal Science and Technology, Sichuan Agricultural University, Chengdu, Sichuan 611130 China; 2 Farm Animal Genetic Resources Exploration and Innovation Key Laboratory of Sichuan Province, Sichuan Agricultural University, Chengdu, Sichuan 611130 China; 3 College of Animal Science and Technology, Henan Agricultural University, Zhengzhou, 450002 Henan China

**Keywords:** Autophagy, Beclin1, Ferroptosis, Follicular atresia, Ubiquitination, USP9X

## Abstract

**Background:**

Follicular atresia, a complex degenerative process regulated by multiple molecular mechanisms, significantly affects female reproductive performance in animals. While granulosa cell (GC) apoptosis has been well established as a primary mechanism underlying follicular atresia, the potential involvement of ferroptosis, which is an iron-dependent form of regulated cell death, remains largely unexplored in chickens.

**Results:**

Using a tamoxifen (TMX)-induced avian model of follicular atresia, we demonstrated that ferroptosis plays a critical role in follicular degeneration. Inhibition of ferroptosis through pharmacological agents significantly restored follicular function, underscoring its potential as a therapeutic target. Notably, we observed a significant upregulation of ubiquitin-specific peptidase 9, X-linked (USP9X) in GCs during atresia. Through comprehensive in vitro and in vivo investigations, we confirmed that USP9X facilitates follicular atresia by promoting ferroptosis in GCs. Mechanistically, USP9X induces ferroptosis by stabilizing Beclin1 through deubiquitination, thereby activating autophagy-dependent ferroptosis. This pathway was effectively suppressed by autophagy inhibitors, emphasizing the essential role of autophagy in USP9X-mediated ferroptosis.

**Conclusions:**

Our findings provide the evidence that the USP9X-Beclin1 axis regulates autophagy-dependent ferroptosis during avian follicular atresia. These insights reveal novel molecular targets and potential genetic markers for improving reproductive efficiency in chicken breeding programs.

**Supplementary Information:**

The online version contains supplementary material available at 10.1186/s40104-025-01269-8.

## Background

Ovarian follicles, originating from the ovaries, are essential structures responsible for the production of both oocytes and hormones [1, 2]. Their development is tightly regulated by a complex network of endocrine, paracrine, and autocrine factors that influence the selection of follicles for ovulation [3]. However, more than 99% of follicles fail to reach the pre-ovulatory stage and instead undergo a degenerative process called atresia [4, 5]. GCs play a critical role in determining the fate of follicles, as their apoptosis occurs prior to that of oocytes and theca cells during atresia [6]. In addition, Moreover, GCs are involved in synthesizing factors essential for follicular growth and maintenance, yet they can also self-destruct through multiple pathways, contributing to follicular atresia [7]. Programmed cell death mechanisms including autophagy [8], apoptosis [9], and necrosis [10] have been shown to play fundamental roles in follicular development and atresia by regulating GC survival and, consequently, follicular progression.

Ferroptosis is a newly characterized form of regulated cell death distinct from apoptosis, autophagy, and necrosis. It is characterized by iron accumulation and lipid peroxidation within the cell [11, 12]. Ferroptosis also suppresses the synthesis of GPXs, leading to reduced antioxidant capacity and increased reactive oxygen species (ROS), which ultimately drive oxidative cell death [13]. A growing body of research has demonstrated that ferroptosis contributes to the onset and progression of numerous diseases [14, 15], making its regulatory proteins promising therapeutic targets [16]. In the context of reproductive biology, follicular atresia has been associated with significantly decreased glutathione (GSH) levels and elevated iron concentrations, suggesting a potential involvement of ferroptosis [17, 18]. Recent findings further support this connection; for instance, Basonuclin1 deficiency was shown to trigger oocyte ferroptosis, resulting in premature follicular activation and increased atresia [19]. These studies indicate a strong link between ferroptosis and follicular atresia, however, the precise molecular mechanisms remain to be fully elucidated.

Ubiquitination and deubiquitination are critical post-translational modifications that regulate protein stability [20]. Certain deubiquitinating enzymes (DUBs), such as OTUB1 and BAP1, have been shown to modulate ferroptosis in human cancers [21, 22]. *USP9X*, a growth-regulated DUB, has been found to accumulate following growth stimulation [23]. Although *USP9X* deficiency inhibits cell proliferation and induces cell death in several models [24, 25], its direct relationship with ferroptosis is still unclear. Emerging evidence suggests that USP9X interacts with key ferroptosis regulators, including P62 [26], and Nrf2 [27], implying a potential role in ferroptotic regulation.

In this study, we uncover a critical role for ferroptosis in regulating follicular atresia. Furthermore, we identify USP9X as a key regulator of redox homeostasis and iron metabolism in GCs. Specifically, USP9X promotes ferroptosis by deubiquitinating Beclin1, thereby contributing to the progression of follicular atresia.

## Methods

### Animals

The animal trials were conducted following the approved research protocol by the Animal Ethics Committee of Sichuan Agricultural University (approval No. 80153/2022). Chickens (Roman layers) were obtained from the poultry breeding farm at Sichuan Agricultural University. The birds were housed individually, following a 14-h light and 10-h dark photoperiod, and were given unrestricted access to commercial feed and water. Individual laying cycles were monitored according to daily oviposition timing. This timing was employed to track individual laying cycles each day.

The birds were randomly assigned to the control group (*n* = 10) and the experimental group (*n* = 10). The control group received subcutaneous treatment with ethanol, whereas the experimental group was administered tamoxifen (TMX) at a dosage of 6 mg/kg body weight, dispensed in a 0.3 mL volume. Treatment was applied daily until a cessation in egg production was noted, at which point all the birds were euthanized. The three largest yellow preovulatory F3–F1 (F3 < F2 < F1) follicles, were rapidly isolated from the ovaries, followed by the extraction of their granulosa layers. The tissue samples were preserved at −80 °C for subsequent western blot analysis and at −20 °C for qPCR or ELISA detection.

To evaluate the role of ferroptosis in follicular atresia, birds received daily intraperitoneal injections of the ferroptosis inducers Erastin (200 mg/kg body weight/d) or iron dextran (100 mg/kg body weight/d). The control group was administered an equivalent volume of PBS. Treatments were conducted each morning and continued until egg laying ceased completely in all erastin- or iron dextran-treated hens. Following treatment, ovaries were promptly collected, weighed, and yellow preovulatory follicles were isolated. The granulosa layers were then dissected from these follicles. However, in birds treated with erastin or iron dextran, the granulosa layers could not be separated due to severe follicular atresia. As a result, the entire follicular wall was used to represent the granulosa cell layer in these cases.

### Cell culture

After isolating the F1, F2, and F3 ovarian follicles from the birds, they were submerged in a PBS solution. Next, in accordance with methods outlined in prior descriptions [28], GCs were extracted from the membrane layer of the follicles above. The cell suspension was then seeded at a density of 1 × 10^6^ cells/well into 24-well culture plates. The cells were cultured in DMEM, supplemented with 10% fetal bovine serum and 0.5% streptomycin, at 37 °C in a humidified atmosphere containing 5% CO_2_. Unless otherwise stated, the cell culture media and supplements were procured from Sigma-Aldrich (St. Louis, MO, USA).

### Plasmids, shRNA, and cell transfections

Adenoviral constructs were generated to express FLAG-tagged USP9X, FLAG-tagged USP9X C471A, and Myc-tagged Beclin1. This was accomplished by cloning their open reading frames (ORFs) with the N-terminal FLAG or Myc sequence into the pCDH-CMV-MCS-EF1α-Puro vector. Plasmids encoding ubiquitin-Lys63 tagged with HA (pRK5-HA-ubiquitin-Lys63) and ubiquitin-Lys48 tagged with HA (pRK5-HA-ubiquitin-Lys48) were obtained from addgene (Cambridge, Massachusetts, USA). Utilizing the QuikChange Mutagenesis Kit from Agilent Technologies, site-directed mutagenesis was performed on USP9X following the manufacturer's instructions. The fidelity of all constructs was confirmed via DNA sequencing. Adenoviral shRNA plasmids designed to target USP9X and nonspecific control shRNA were obtained from Genomeditech (Shanghai, China). All transfections were conducted using Lipofectamine 3000 (Invitrogen), following the protocol provided by the manufacturer.

### Adenoviral infection

GC cells were seeded onto six-well plates (5 × 10^6^ cells/well). After 24 h, cells were infected with adenovirus shRNA-control (Ad-shCtrl), adenovirus shRNA-USP9X (pAdEasy-U6-shUSP9X-CMV-eRFP), or adenovirus USP9X overexpression vector (pAdEasy-EF1-USP9X-MCS-3FLAG-CMV-eRFP), all at an MOI of 10.

To increase exogenous expression of USP9X in vivo, pAdEasy-EF1-USP9X-MCS-3FLAG-CMV-eRFP (ad-USP9X) was used, and pAdEasy-EF1-MCS-3FLAG-CMV-eRFP (Ad-Ctrl) was used as control vector. The pAdEasy-CMV-eRFP vector was diluted in 0.9% sodium chloride to a final volume of 1 mL with a final titer of 1 × 10^12^ viral genomes, and then injected into each bird through intraperitoneal injection. Adenovirus interference efficiency was detected by immunofluorescence and western blot.

### Hematoxylin and eosin staining

Hematoxylin and eosin (H&E) staining was performed to visualize the microstructure of the walls of atretic yellow follicles, following established protocols [29]. After sealing the slices with neutral gum, images were captured using an inverted microscope (Leica, Hesse, Germany).

### RNA isolation and real-time PCR

Total RNA extraction from yellow follicles and cells was accomplished using a total RNA isolation kit (Foregene, Chengdu, China), adhering to the guidelines provided by the manufacturer. The PrimeScript™ RT reagent Kit (Takara, Tokyo, Japan) was used for cDNA synthesis, and real-time quantitative PCR (qPCR) analysis was conducted with SYBR^®^ Premix Ex Taq Ⅱ (Takara), following the manufacturer's guidelines. Three biological replicates were performed for each experiment, and the qPCR results were analyzed using the ΔCt method to normalize the period threshold [30]. The oligonucleotide primers utilized in this study are listed in Table S1.

### Double-labeled adenovirus mRFP-GFP-LC3 transfection

Adenoviruses synthesizing the mRFP-GFP-LC3 fusion protein (HanBio, Shanghai, China) were employed to infect GC cells. After 24 h, the cells were washed thrice with PBS and fixed in 4% paraformaldehyde. Subsequently, they were stained with DAPI to visualize the nuclei. The images were captured using the LSM 510 confocal microscope (Zeiss, Oberkochen, Germany).

### Western blot assay and immunoprecipitation assay

Total protein was extracted from yellow follicles and cells using cell lysis buffer (Cell Signaling Technology) with added phosphatase inhibitor (Sigma-Aldrich) and protease inhibitor (Promega Madison, Wisconsin, USA). The quantification of protein concentration was performed utilizing the Bradford assay (Bio-Rad, Hercules, CA, USA). About 20 μg of protein were electrophoresed on SDS-PAGE gels and transferred to a PVDF membrane. Molecular weight was determined using a kaleidoscope prestaining standard (Sigma-Aldrich). After blocking the PVDF membrane with 5% milk, it was incubated with the primary antibody overnight at 4 °C. The following day, PVDF membranes were incubated with HRP-labeled secondary antibodies for 1 h, then treated with enhanced chemiluminescence reagent (Millipore, Bedford, MA, USA). Densitometric analysis of the bands was conducted using ImageJ. HEK293T or GC cells were lysed with an IP lysis solution (Sigma-Aldrich) containing phosphatase inhibitors for the immunoprecipitation analysis. Total protein was obtained through immunoprecipitation using FLAG, HA, and Myc-tag antibodies in conjunction with protein A/G beads. After washing three times with IP lysis buffer, the immunocomplexes were analyzed using western blotting techniques.

### Antibodies

The following primary antibodies were used: mouse anti-USP9X (cat. no. sab2107379, Sigma-Aldrich), Rabbit anti-Beclin1 (cat. no. ab62577, Abcam), Rabbit anti-p62 (cat. no. 232145, Cell Signaling Technology), mouse anti-GPX4 (cat. no. ab252833, Abcam), anti-ACSL4 (cat. no. sab2100035, Sigma-Aldrich), rabbit anti-FTH1 (cat. no. 43935, Cell Signaling Technology), rabbit anti-ubiquitin (cat. no. ab134953, Abcam), mouse anti-Myc-tag (cat. no. 05-724, Sigma), mouse anti-FLAG-tag (cat. no. F3165, Sigma-Aldrich), rabbit anti-HA-tag (cat. no. ab9110, Abcam), mouse anti-GAPDH (cat. no. AB2302, Sigma-Aldrich). The following secondary antibodies were used: goat anti-rabbit Alexa Fluor^®^ 594 (cat. no. 88895, Cell Signaling Technology), donkey anti-rabbit Alexa Fluor^®^ 488 (cat. no. ab150073, Abcam), goat anti-mouse HRP (cat. no. sc2005, Santa Cruz Biotechnology), rabbit anti-mouse HRP (cat. no. ab6728, Abcam), goat anti-rabbit HRP (cat. no. AP510, Sigma-Aldrich).

### Immunofluorescence and confocal microscopy

After a 15-min fixation in 4% paraformaldehyde, the cells underwent a series of three washes using PBS. They were subsequently permeabilized with 0.5% Triton X-100 for 5 min. The specimens were overnight incubated at 4 °C with the primary antibody, suitably diluted in PBS containing 1% BSA. Two consecutive 10 min PBS rinses followed this. After primary antibody incubation and washing, the samples were exposed to appropriate fluorescently labeled secondary antibodies for 1 h in the dark. Fluorescence intensity was then observed under a TCS SP5 confocal microscope (Leica Wezler, Germany).

### Lipid peroxidation, GSH, and iron assay

Cells were cultured in 12-well plates, and after specific treatment, cell lysates were obtained by centrifugation. The MDA (cat. no. ab118970, Abcam) or GSSG (cat. no. ab141393, Abcam) lipid peroxidation detection kits were used by the manufacturer's instructions to measure lipid peroxidation product levels in the cell lysates. GSH concentration levels were quantified with a GSH assay kit (cat. no. CS0260; Sigma-Aldrich), and ferrous iron concentration was determined using an iron assay kit (cat. no. ab83366, Abcam).

### Analysis of Beclin1 deubiquitylation

To conduct the in vivo Beclin1 ubiquitylation assay, cells were transfected with the designated plasmids and exposed to 20 μmol/L MG132 for 12 h. Subsequently, the samples were harvested and subjected to lysis using NETN buffer (Sigma) supplemented with 0.1% SDS, 20 μmol/L MG132, and a cocktail of protease inhibitors. The lysates were incubated with an anti-Beclin1 antibody for 3 h and protein A/G agarose beads for an additional 8 h at 4 ℃. The precipitated proteins were liberated from the beads through a boiling procedure lasting 10 min in the SDS-PAGE loading buffer. Subsequently, these proteins were subjected to immunoblotting (IB) using the anti-HA antibody.

### Transmission electron microscopy (TEM)

Cells were cultured in 6-well plates and carefully scraped off following special treatment. After two PBS washes, the cells were fixed in 2% glutaraldehyde overnight at 4 ℃ and then treated with 2% osmium tetroxide for 2 h. After washing three times with PBS, they were stained with 2% uranyl acetate at room temperature for 1 h and embedded in 3% agarose. Following dehydration through a series of acetone solutions (50%, 70%, 90%, and 100%), the samples were sliced using the Leica EM UC7 (Leica, Hesse, Germany) and examined under a Hitachi HT7700 (Hitachi, Tokyo, Japan).

### Cell viability assay

Cell death was assessed by staining with propidium iodide (PI; cat. no. S6874, Selleck) according to the manufacturer's protocol, followed by fluorescence microscopy observation. Cell viability was measured using the Cell Counting Kit-8 (CCK8). After undergoing specific treatment, cells were seeded into 96-well plates. Then, 10 μL of CCK8 was added to each well and incubated for 2 h. The live cell count was determined by measuring the absorbance at 450 nm using a Varioskan LUX Elisa (ThermoFisher, Waltham, MA, USA).

### Adenoviral and adeno-associated virus infection

GCs were cultured in 6-well plates, followed by transfection with various adenoviruses, including shRNA-control (Ad-shCtrl), adenovirus-USP9X-shRNA, adenovirus-shRNA-Beclin1, adenovirus vector (Ad-Vec), Ad-USP9X, Ad-Beclin1, or Ad-Atg7-shRNA, following the manufacturer's instructions. All adenovirus vectors were purchased from HanBio Technology Co., Ltd. (Shanghai, China).

### Statistical analysis

The results were expressed as means ± SD (standard deviation). A student's paired *t*-test was used to compare the means of two groups, while a one-way analysis of variance (ANOVA) was employed for comparisons among more than two groups. Post hoc comparisons of group differences were made using the least significant difference test when the ANOVA yielded significant results. Statistical calculations were performed using graphPad prism 6.0 software, with statistical significance defined as a *P*-value less than 0.05.

## Results

### Ferroptosis induces follicular atresia by upregulating USP9X expression

**Fig. 1:**
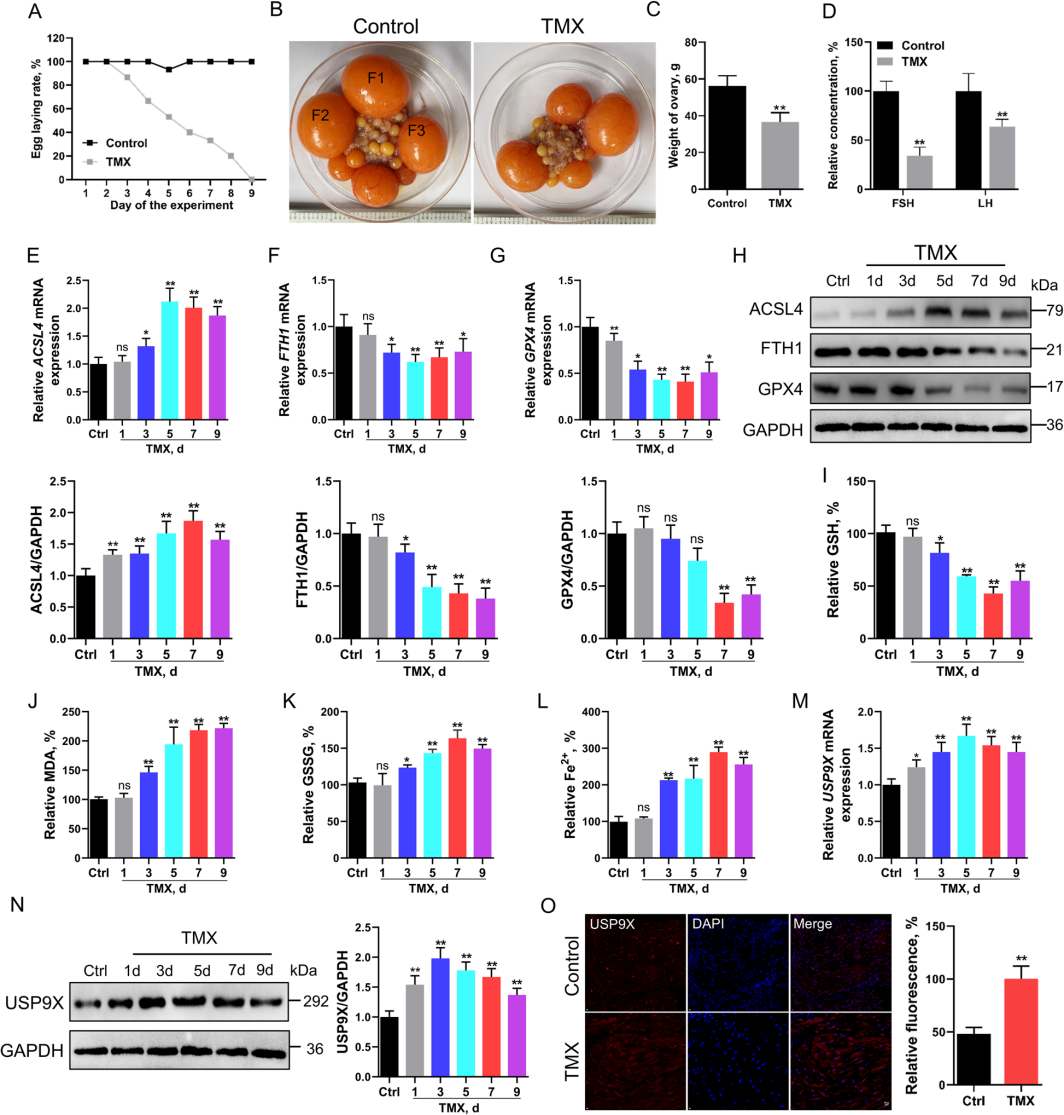
Induction of ferroptosis during follicular atresia and increased expression of USP9X. **A**–**C** Assessment of egg-laying rate, ovary morphology, and ovarian weights in birds (*n* = 12) following treatment with tamoxifen (TMX) or vehicle (ethanol; control). The three most prominent yellow preovulatory follicles, F3–F1 (F3 < F2 < F1), are illustrated. In TMX-treated birds, there is a noticeable reduction in the number and size of large yellow follicles compared to the control group. **D** Depiction of follicle-stimulating hormone (FSH) and luteinizing hormone (LH) concentrations in blood plasma from birds exposed to TMX for 9 d (*n* = 6). **E**–**G** qPCR analysis representing the expression levels of *ACSL4*, *FTH1*, and *GPX4* mRNA in bird follicles during treatment with TMX (*n* = 6). **H** Western blot evaluation of ACSL4, FTH1, and GPX4 protein levels in bird follicles during TMX treatment (*n* = 3), with GAPDH as a loading control. **I**–**L** Graphs illustrating the relative concentrations of GSH, MDA, iron content, and glutathione disulfide (GSSG) in bird follicles throughout TMX treatment (*n* = 6). **M** The qPCR assessment of *USP9X* mRNA expression in bird follicles during TMX intervention (*n* = 6). **N** Western blot analysis highlighting USP9X protein expression levels in bird follicles following TMX treatment (*n* = 3), utilizing GAPDH as a loading standard. **O** Immunofluorescence imagery reveals the presence of USP9X in bird follicles subjected to TMX or vehicle over 9 d (*n* = 3). Error bars represent the means ± SD. ^*^P < 0.05, ^**^P < 0.01. Non-significant differences were denoted with the notation "ns"

To investigate the role of ferroptosis in follicular atresia, a TMX-induced avian model was established. As expected, TMX-treated birds exhibited complete cessation of oviposition by day 9 (Fig. 1A) and showed a 34% reduction in ovarian weight compared to controls (Fig. 1B and C). Serum levels of follicle-stimulating hormone (FSH) and luteinizing hormone (LH) were significantly reduced (*P* < 0.05) in the TMX group (Fig. 1D). TMX treatment increased the expression of ACSL4, a ferroptosis marker, while reducing GPX4 and FTH1 levels (Fig. 1E–H). In addition, TMX led to decreased GSH levels, and significant increases in MDA, GSSG, and intracellular iron concentrations (*P* < 0.01; Fig. 1I–L), collectively indicating the activation of ferroptosis in the atretic follicles.

To further investigate the regulatory mechanism underlying ferroptosis in follicular atresia, RNA sequencing (RNA-Seq) of the granulosa layer of healthy and atretic follicles was conducted. The expression of ferroptosis pathway receptors and downstream target genes was significantly upregulated in atretic follicles, whereas folliculogenesis was significantly decreased (*P* < 0.05; Fig. S1A). Interestingly, USP9X expression, which has been strongly linked to female infertility, was significantly upregulated in the atretic follicles (Fig. S1B and C). In addition, qPCR and western blot analysis revealed a significant increase in USP9X expression following TMX treatment (*P* < 0.01; Fig. 1M and N). Similarly, immunofluorescence staining revealed an upregulation in USP9X expression in atretic follicles (Fig. 1O). KEGG pathway analysis demonstrated that TMX treatment resulted in the regulation of 15 signaling pathways, including those involved in ferroptosis and autophagy (Fig. S1D). This implies that ferroptosis may be involved in the regulation of follicular atresia via the action of USP9X.

### Ferroptosis-induced GC death results in follicular atresia

**Fig. 2:**
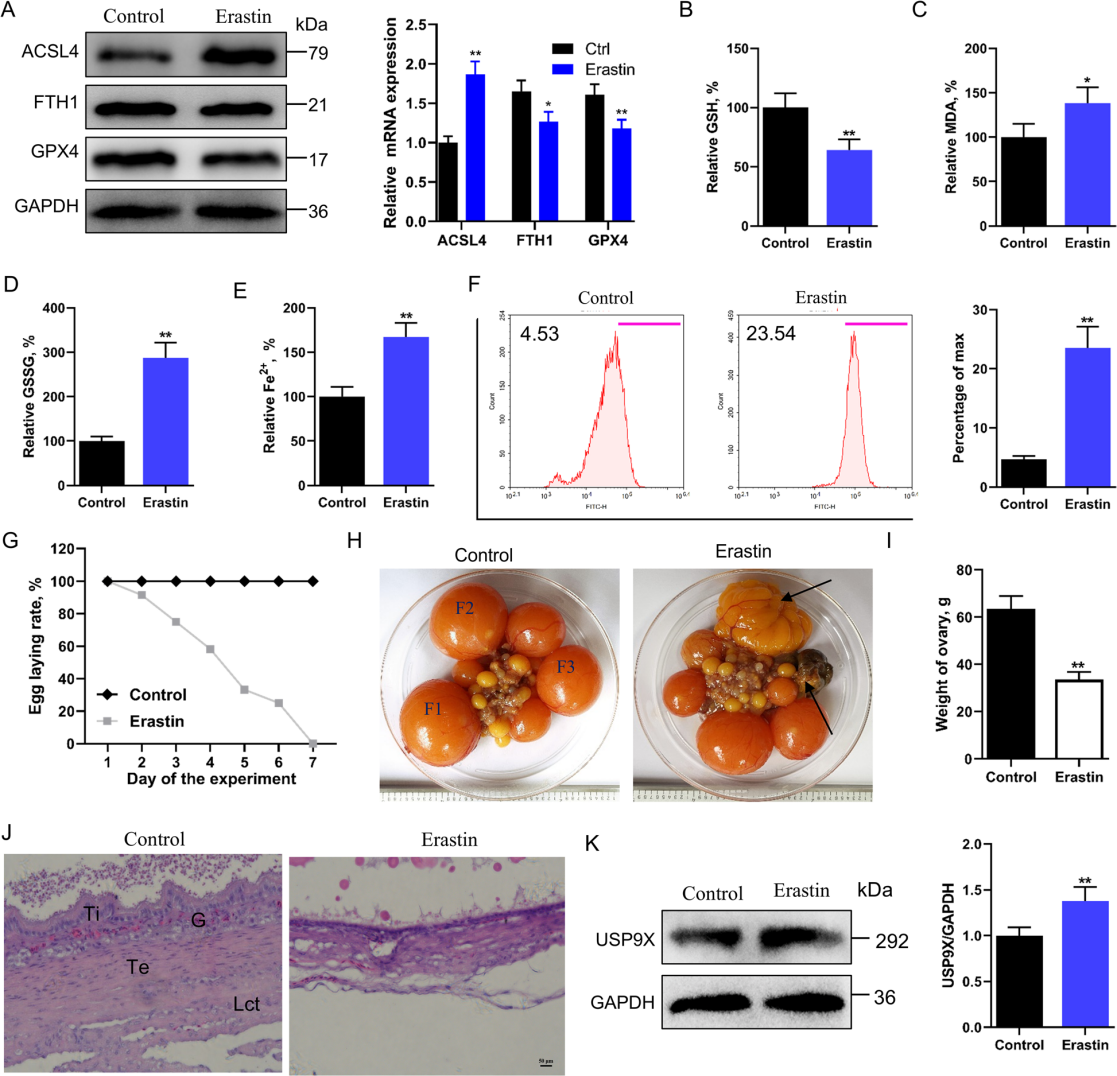
Potential induction of follicular atresia through ferroptosis. **A** Western blot analysis of ACSL4, FTH1, and GPX4 protein expression levels in bird follicles post-Erastin treatment (*n* = 3), with GAPDH as a loading control. **B**–**E** Graphical representation of the relative GSH, MDA, iron content, and GSSG concentrations in bird follicles following erastin exposure (*n* = 6). **F** Flow cytometry assessment of lipid peroxidation levels in bird follicles post-erastin treatment (*n* = 6). **G**–**I** Examine egg-laying rate, ovary morphology, and ovary weights in bird follicles after erastin treatment (*n* = 10). F3–F1 (F3 < F2 < F1) indicates the three most prominent yellow preovulatory follicles. **J** Hematoxylin and eosin (H&E) staining of bird follicles after erastin or vehicle administration (*n* = 6), illustrating G: granulosa layer; Ti: theca interna layer; Te: theca externa layer; Lct: loose connective tissue and epithelium. **K** Western blot determination of USP9X protein expression level in bird follicles following Erastin treatment (*n* = 3). Error bars represent the means ± SD. ^*^P < 0.05, ^**^P < 0.01

To further validate the involvement of ferroptosis, birds were administered erastin, a known ferroptosis inducer. Erastin treatment significantly activated ferroptotic processes (*P* < 0.01; Fig. 2A–F) and led to complete oviposition cessation by 7 d (Fig. 2G). Ovarian weight decreased by 47%, and histological analysis revealed extensive hemorrhage and follicular atresia in yellow hierarchical follicles (Fig. 2H and I). In addition, intraperitoneal injection of irondextran was utilized to induce iron accumulation, and we found that irondextran treatment triggered ferroptosis, ultimately leading to follicular atresia (Fig. S2A–I). The histology of healthy and erastin-treated yellow preovulation follicle walls is shown in Fig. 2J. Erastin-treated birds exhibited significant atretic changes in the largest yellow follicular walls, including the disappearance of the granular layer and basement membrane, compared with control birds. Thus, erastin may induce follicular atresia by promoting ferroptosis in GCs. In addition, erastin treatment significantly upregulated USP9X expression (*P* < 0.01; Fig. 2K). This suggests that USP9X may regulate ferroptosis in GCs to mediate the process of follicular atresia.

**Fig. 3:**
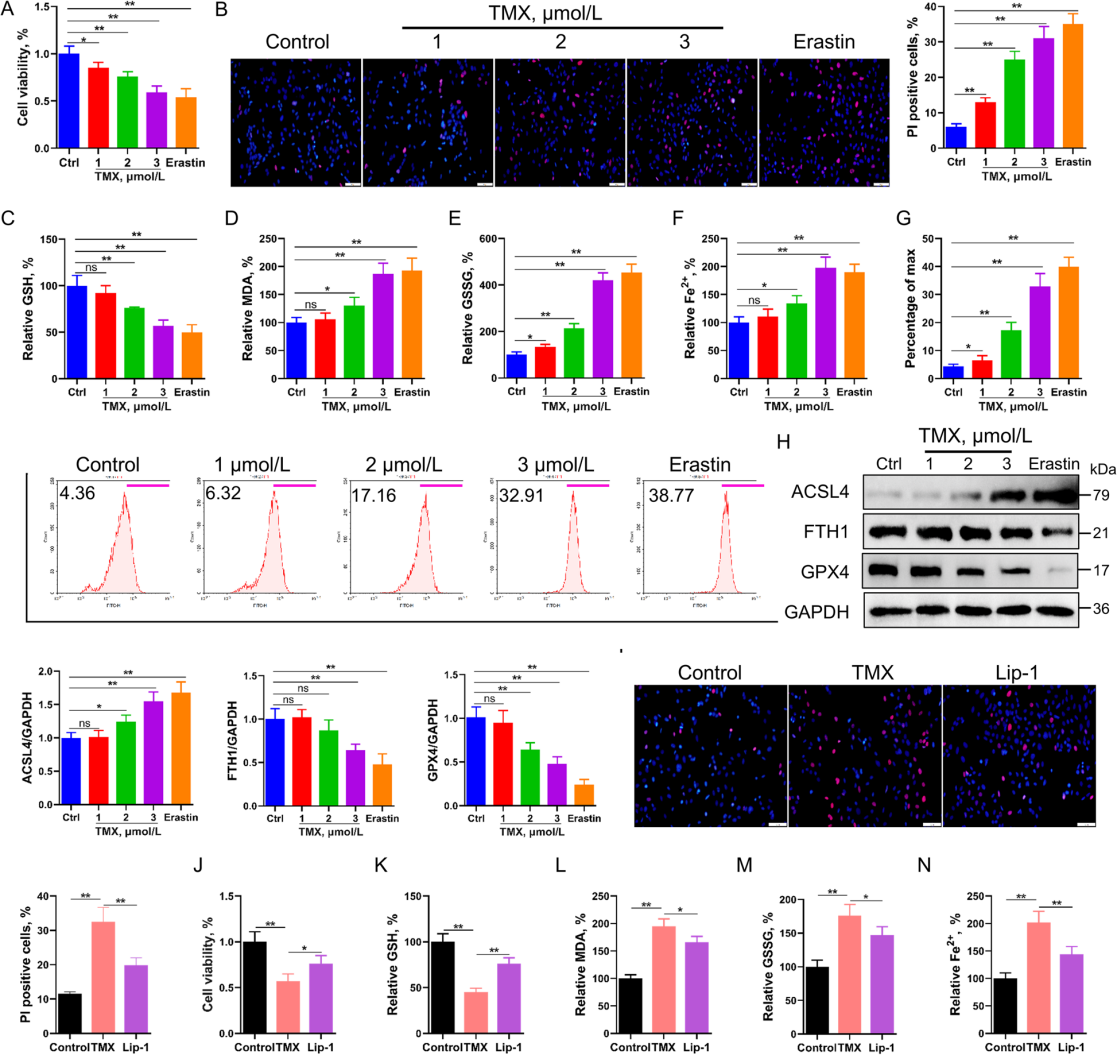
Induction of ferroptosis in granulosa cells (GCs) by TMX treatment. **A** Cell viability assessment employing the CCK-8 assay after treatment with TMX or erastin in GCs (*n* = 6). **B** Utilization of propidium iodide (PI) staining to evaluate cell viability in GCs following TMX or erastin treatment (*n* = 6). **C–F** Graphs presenting the relative concentrations of GSH, MDA, iron content, and GSSG in GCs after exposure to TMX or erastin (*n* = 6). **G** Flow cytometry analysis of lipid peroxidation levels in GCs following treatment with TMX or erastin (*n* = 6). **H** Western blot exploration of ACSL4, FTH1, and GPX4 protein levels in GCs after erastin application (*n* = 3), with GAPDH as a loading control. **I** and **J** GCs were preincubated with lipoxstatin-1 (100 nmol/L) for 12 h, followed by a 2-h TMX treatment. CCK8 and PI staining were used to detect cell viability (*n* = 6). **K**–**N** Following 12 h preincubation with lipoxstatin-1 and a subsequent 2 h TMX treatment, GSH, MDA, GSSG, and iron concentrations were determined in GCs (*n* = 6). Error bars represent the means ± SD. ^*^P < 0.05, ^**^P < 0.01. Non-significant differences were denoted with the notation "ns"

To investigate the potential occurrence of ferroptosis in GCs, the cells were exposed to three ferroptosis inducers: erastin, sorafenib, and RSL3. Results revealed a significant induction of ferroptosis following treatment with all three inducers. Notably, the ferroptosis inhibitor liproxstatin-1 (lip-1) significantly impaired the classical ferroptosis events in GCs; however, no such effect was observed with the necroptosis inhibitor necrostatin-1 or the apoptosis inhibitor ZVD-FMK (*P* < 0.05; Fig. S3A–F). We then investigated whether TMX could induce follicular atresia in GCs by triggering ferroptosis. The results showed that treatment of GCs with erastin or TMX significantly decreased cell viability, as demonstrated by CCK8 and PI staining (*P* < 0.01; Fig. 3A and B). In addition, GSH depletion, lipid peroxidation accumulation, and iron overload were observed, indicating that TMX or erastin treatment can induce ferroptosis in GCs (Fig. 3C–G). Moreover, treatment with TMX and erastin led to a marked upregulation of ACSL4, a key promoter of ferroptosis, while significantly reducing the expression levels of GPX4 and FTH1, both of which are critical ferroptosis suppressors (Fig. 3H). To evaluate the therapeutic potential of ferroptosis inhibition, birds with TMX-induced follicular atresia were subsequently treated with lip-1, a potent and selective ferroptosis inhibitor, in both in vivo and in vitro models. Results from the CCK-8 assay and PI staining demonstrated that lip-1 effectively reduced granulosa cell death (Fig. 3I and J). Furthermore, lip-1 significantly suppressed TMX-induced ferroptosis (*P* < 0.05; Fig. 3K–N, Fig. S4A–I). Notably, by 9 d post-TMX treatment, the affected birds exhibited a complete cessation of egg production. However, subsequent administration of lip-1 resulted in a sustained egg production rate exceeding 50% (Fig. S4J). Furthermore, treatment with lip-1 significantly increased ovarian weight, and the yellow follicles displayed well-defined hierarchical organization. Notably, no signs of hemorrhage or atresia were observed in the follicles (Fig. S4K and L). Collectively, these findings indicate that granulosa cell ferroptosis contributes to follicular atresia, whereas inhibition of ferroptosis supports the restoration of normal follicular structure and function.

### USP9X induces follicular atresia by mediating ferroptosis in GCs

**Fig. 4:**
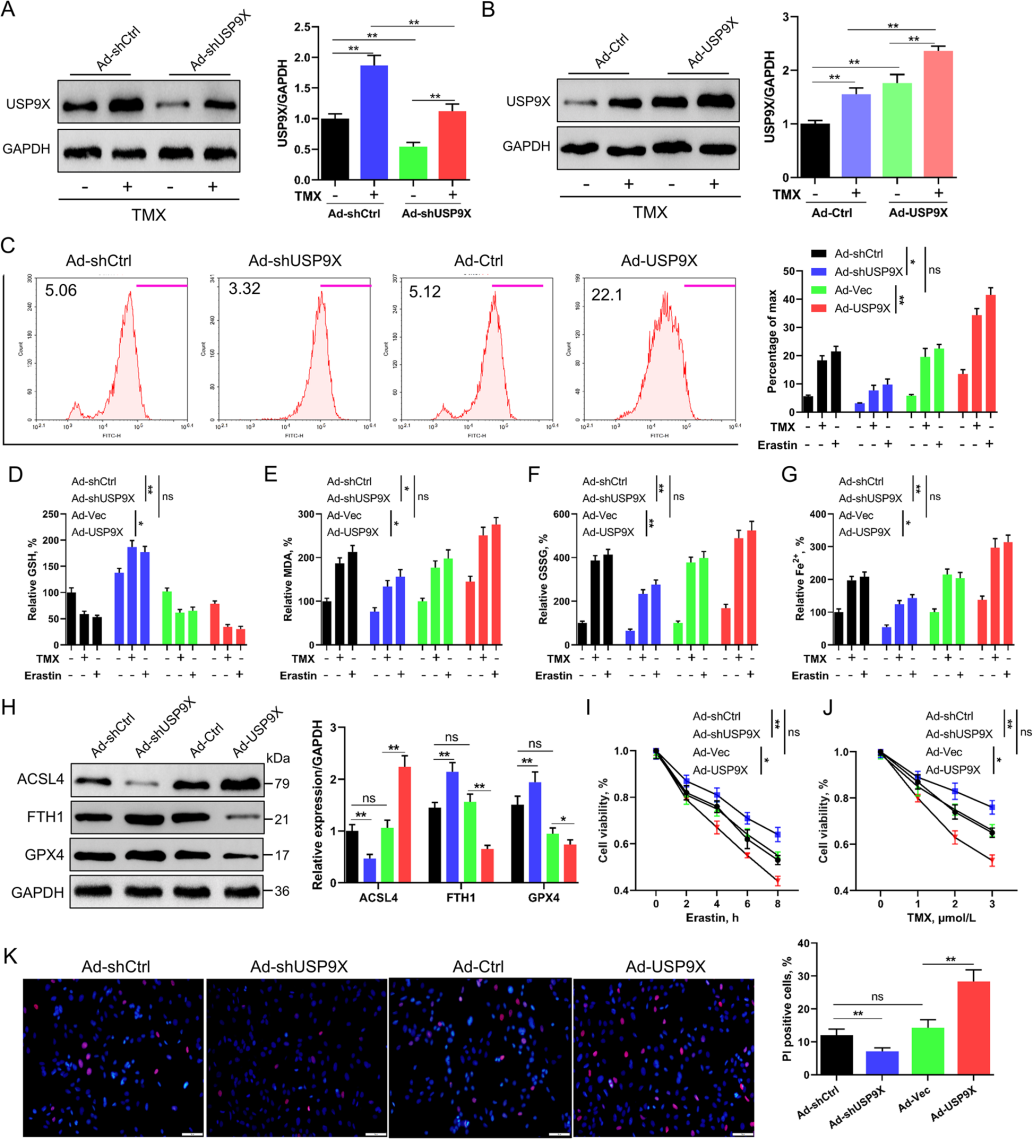
Role of USP9X in modulating ferroptosis in GCs. GCs were transfected with Ad-shUSP9X or Ad-USP9X, then treated with erastin (5 μmol/L) for 6 h or TMX (2 μmol/L) for 24 h. **A** and **B** Western blot validation of the transfection efficiency of USP9X (*n* = 3), with GAPDH as a loading control. **C–G** Assessment of lipid peroxidation and GSH, MDA, GSSG, and iron concentrations in GCs with USP9X silencing or overexpression (*n* = 6). **H** Western blot exploration of ACSL4, FTH1, and GPX4 protein expression levels in USP9X-silenced or overexpressed GCs (*n* = 3), utilizing GAPDH as a loading control. **I** and **J** Utilization of the CCK-8 assay to gauge cell viability under erastin or TMX treatment conditions in GCs with manipulated USP9X expression (*n* = 6). **K** Propidium iodide PI staining was employed to evaluate cell survival in GCs with USP9X silencing or overexpression (*n* = 6). Error bars represent the means ± SD. ^*^P < 0.05, ^**^P < 0.01. Non-significant differences were denoted with the notation "ns"

To investigate whether the upregulation of USP9X by TMX or erastin treatment directly contributes to the induction of ferroptosis, GCs were pretreated with Ad-shUSP9X or Ad-USP9X. Western blotting confirmed that USP9X protein levels were significantly decreased in ad-shUSP9X-treated cells, whereas they were significantly increased in Ad-USP9X-treated cells (*P* < 0.01; Fig. 4A and B). Furthermore, overexpression of USP9X significantly exacerbated ferroptosis, while silencing USP9X mitigated ferroptotic responses, regardless of whether the cells were exposed to erastin or TMX (Fig. 4C–H). Specifically, knockdown of USP9X reduced the vulnerability of granulosa cells to TMX- or erastin-induced cell death, whereas USP9X overexpression markedly enhanced their sensitivity to ferroptotic damage (Fig. 4I and J). PI staining further confirmed that USP9X knockdown exerted a protective effect on cell viability, whereas USP9X upregulation decreased cell viability (Fig. 4K). As high levels of erastin have been shown to induce apoptosis, we investigated whether treating GCs with erastin activated the apoptotic pathways. For this, GCs were treated with erastin for 24 h, with or without the ferroptosis inhibitors (lip-1) or iron chelator (DFX). We observed that either lip-1 or DFX was effective in preventing erastin-induced ferroptosis (Fig. S5A–G). Collectively, these findings suggest that USP9X positively regulates ferroptosis in GCs.

To further elucidate the role of USP9X in granulosa cell-mediated follicular atresia, birds were administered an adenoviral (Ad)-based vector engineered to overexpress USP9X. Following Ad-USP9X treatment, a marked upregulation of USP9X expression was observed in the granulosa layer of follicles in both untreated control birds and those subjected to TMX-induced atresia (Fig. S6A and B). Additionally, immunofluorescence analysis confirmed effective transduction of follicular tissue by the Ad-USP9X construct, with statistically significant infection efficiency (*P* < 0.05; Fig. S6C). In addition, birds treated with Ad-USP9X had significantly higher levels of ferroptosis in the follicular granular layer (*P* < 0.05; Fig. S6D–F). However, there was no difference in the egg laying rate and ovarian morphology between Ad-USP9X treated birds and control birds (data not shown); however, Ad-USP9X was found to accelerate follicular atresia in the presence of TMX (Fig. S6G). These results suggest that USP9X plays a key role in promoting ferroptosis in GCs, ultimately resulting in follicular atresia.

### USP9X-mediated ferroptosis is associated with autophagy activation

**Fig. 5:**
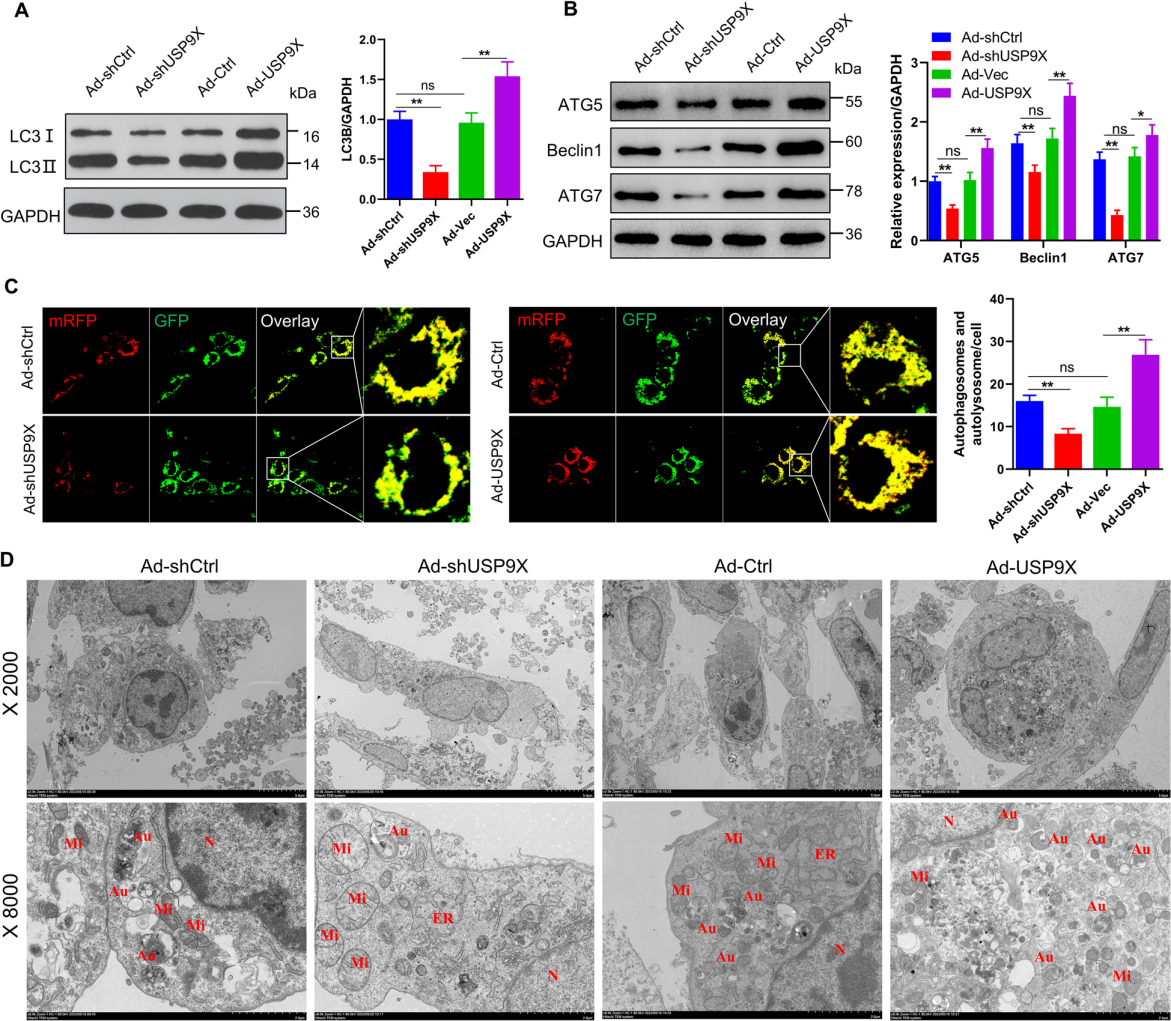
USP9X-induced ferroptosis is linked to autophagy activation. **A** Western blotting was used to measure LC3B protein expression levels in GCs with silenced or overexpressed USP9X (*n* = 3), with GAPDH as a loading control. **B** Western blot assessment of ATG5, ATG7, and Beclin1 protein levels in GCs with manipulated USP9X expression (*n* = 3), employing GAPDH as a loading control. **C** Confocal microscopy to quantify autophagosomes and autolysosomes in GCs with USP9X silencing or overexpression following transfection with mRFP-GFP-LC3 adenovirus (*n* = 3). **D** Electron microscopy examined ultrastructure changes in GCs after USP9X knockdown or overexpression (*n* = 3), highlighting Mi: Mitochondria; Au: Autophagosome; ER: Endoplasmic reticulum; N: Nucleus. Error bars represent the means ± SD. ^*^P < 0.05, ^**^P < 0.01. Non-significant differences were denoted with the notation "ns"

Ferroptosis is a type of programmed cell death that involves autophagy [31]. To determine whether USP9X regulates ferroptosis through modulation of the autophagy pathway, we conducted a series of molecular analyses. Western blotting demonstrated that silencing USP9X led to a reduction in LC3B expression, whereas its overexpression resulted in a marked increase in LC3B levels (Fig. 5A). Furthermore, USP9X overexpression significantly elevated the expression of key autophagy-related proteins, including Beclin-1, ATG5, and ATG7, while USP9X knockdown caused a significant downregulation of these proteins (*P* < 0.01; Fig. 5B). To further assess autophagic activity, granulosa cells were transfected with mRFP-GFP-LC3 AAV constructs, allowing real-time visualization and monitoring of autophagic flux. The results showed that Ad-shUSP9X reduces autophagosome levels, whereas pretreatment with ad-USP9X significantly increased their levels (*P* < 0.01; Fig. 5C). Moreover, transmission electron microscopy revealed reduced and increased number of autophagosomes following treatment with Ad-shUSP9X and ad-USP9X, respectively (Fig. 5D).

Furthermore, to investigate if USP9X regulates autophagy-mediated ferroptosis, we inhibited autophagy using the autophagy inhibitor chloroquine (CQ) or through ATG7 knockdown (Fig. S7A and G). CQ treatment or ATG7 knockdown reduced ACSL4 levels and increased GPX4 and FTH1 levels, regardless of USP9X treatment (Fig. S7B and H). Furthermore, under conditions of ATG7 knockdown or CQ treatment both of which inhibit autophagy Ad-USP9X treatment resulted in a noticeable reduction in ferroptosis activity. Importantly, suppression of autophagy through either genetic silencing of ATG7 or pharmacological inhibition with CQ effectively reversed USP9X-induced ferroptosis in granulosa cells (Fig. S7C–F and S7I–L). These findings strongly indicate that USP9X mediates ferroptosis through an autophagy-dependent mechanism, thereby contributing to the regulation of follicular atresia.

### USP9X regulates autophagy dependent-ferroptosis by stabilizing Beclin1

**Fig. 6:**
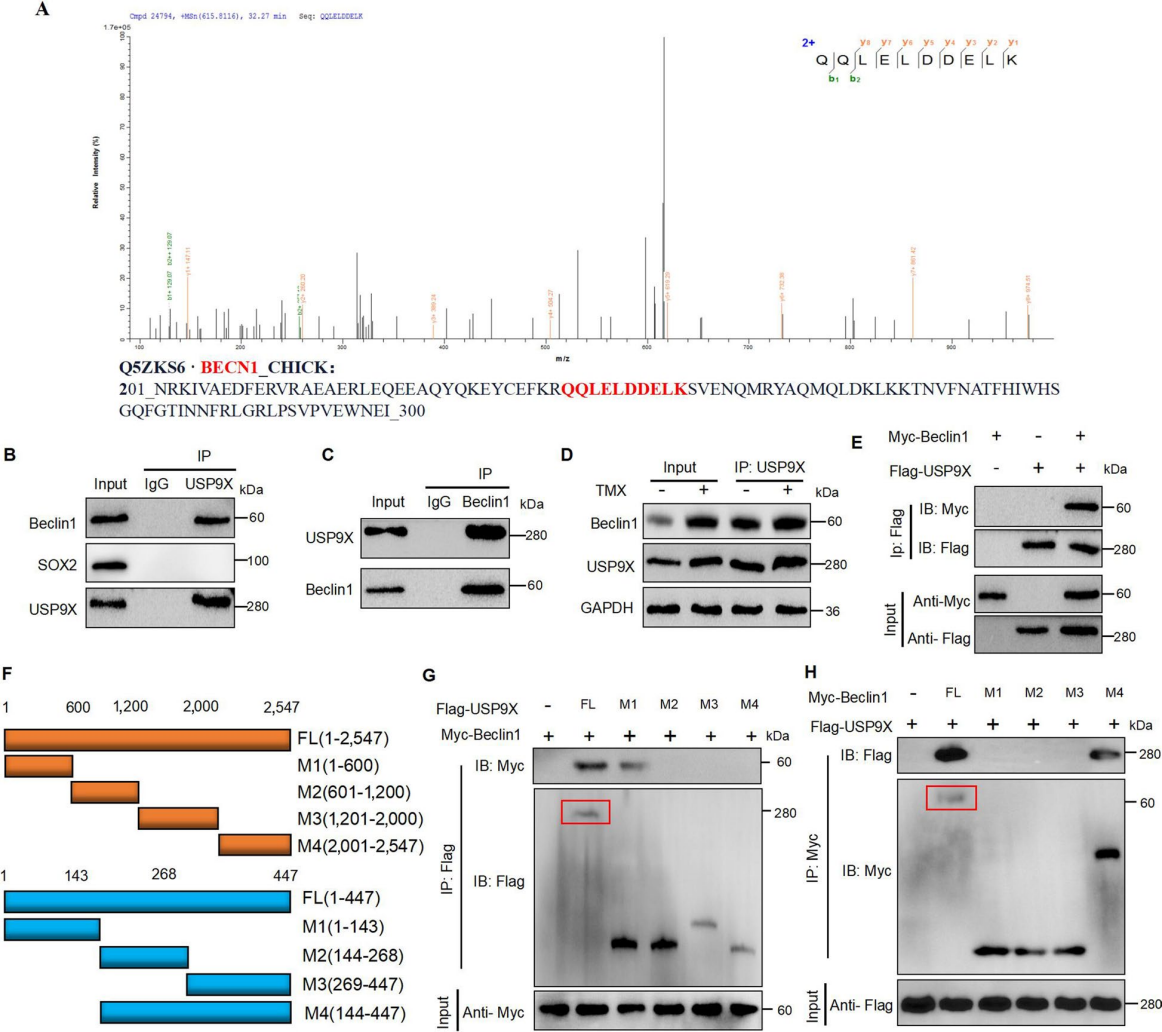
Interaction between USP9X and Beclin1 in regulating autophagy-dependent ferroptosis. **A** IP/MS analysis identifying Beclin1 as a protein interacting with USP9X. **B** Evaluation of protein binding involving endogenous levels in GCs lysate was conducted through immunoprecipitation utilizing either anti-USP9X or anti-IgG antibodies. The presence of Beclin1 and SOX2 was determined using the designated antibodies through immunoblotting (*n* = 3). **C** Execution of reciprocal co-immunoprecipitation analysis to explore the interaction between USP9X and Beclin1 in GCs (*n* = 3). **D** Post-12 h treatment with TMX in vitro, an equal amount of protein lysate was exposed to immunoprecipitation employing anti-USP9X antibodies, followed by usage alongside the indicator (*n* = 3). **E** Performance of reciprocal co-immunoprecipitation analysis between Myc-labeled Beclin1 and FLAG-labeled USP9X in HEK 293T cells. IB denotes immunoblotting, and IP represents immunoprecipitation. **F** Schematic illustrations of FLAG-tagged full-length (FL) USP9X and Myc-tagged FL Beclin1, along with various deletion mutants. **G** Co-transfection of Myc-Beclin1 and FLAG-tagged FL USP9X or its deletion mutants into HEK 293T cells, followed by examination of cell lysates through IP with FLAG beads and IB with Myc and FLAG antibodies (*n* = 3). **H** Co-transfection of FL Beclin1 or its deletion mutant with FLAG-USP9X and Myc-labeled FL Beclin1 in HEK 293T cells facilitated the analysis of the cell lysate by immunoprecipitation. Anti-FLAG and anti-Myc antibodies were employed in the subsequent immunoblot study (*n* = 3). Error bars represent the means ± SD. ^*^P < 0.05, ^**^P < 0.01

To study the mechanism by which USP9X regulates autophagy-dependent ferroptosis, immunoprecipitation tandem mass spectrometry (IP/MS) was used to investigate the proteins that bind to USP9X. The results identified Beclin1 as a potential interacting protein of USP9X (Fig. 6A). Moreover, co-immunoprecipitation (Co-IP) analysis validated the IP/MS results, demonstrating that endogenous USP9X directly interacted with Beclin1 in GCs, whereas SOX2 (another protein known to bind to USP9X and regulate ferroptosis) or control IgG failed to co-precipitate (Fig. 6B). In addition, results of reverse Co-IP analysis demonstrated significant precipitation of USP9X by Beclin1 in GCs (*P* < 0.05; Fig. 6C). Moreover, TMX was found to facilitate the interaction between USP9X and Beclin1 (Fig. 6D). A Co-IP with Myc-labeled Beclin and FLAG-Nestin-labeled proteins in HEK 293 T cells showed that Myc-labeled Beclin1 and FLAG-labeled USP9X efficiently co-precipitated in HEK 293 T cells (Fig. 6E). To delineate the specific binding regions between USP9X and Beclin1, we employed full-length and truncated constructs of FLAG-tagged USP9X and Myc-tagged Beclin1 in co-immunoprecipitation assays using HEK 293T cells (Fig. 6F). The analysis revealed that the C-terminal region of USP9X, specifically amino acid residues 1–600, was essential for its interaction with Beclin1 (Fig. 6G). In addition, USP9X selectively bound to the combined M2 and M3 domains of Beclin1, whereas no interaction was observed when M1, M2, or M3 domains were expressed individually (Fig. 6H). This finding suggested that the interaction between USP9X and Beclin1 may be dependent on the linkage region connecting the M2 and M3 domains, USP9X may interact with Beclin1 to regulate autophagy-dependent ferroptosis.

### USP9X stabilizes Beclin1 by deubiquitination modifications

**Fig. 7:**
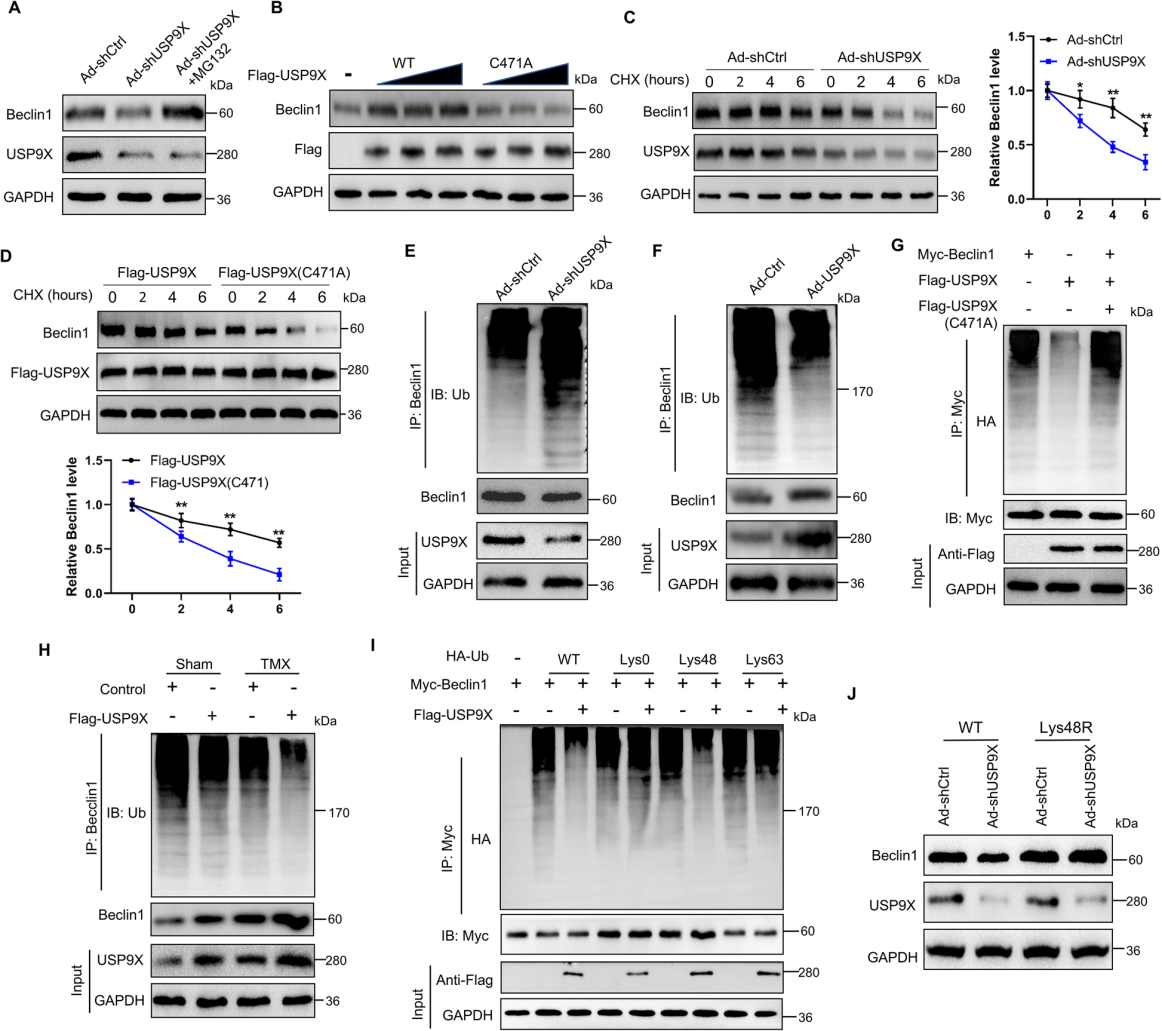
Stabilization of Beclin1 expression through USP9X-mediated deubiquitination. **A** Western bolt analysis of Beclin1 and USP9X expression in Ad-shUSP9X GCs treated with or without the proteasome inhibitor MG132 (*n* = 3). GAPDH served as a loading control. **B** HEK 293 T cells were transfected with increasing quantities of FLAG-tagged USP9X (WT or C417A mutant). Subsequently, the cellular lysate was subjected to immunoblotting analysis utilizing either anti-Beclin1 or anti-FLAG antibodies (*n* = 3). **C** Western blot analysis of Beclin1 protein level in Ad-shCtrl and Ad-shUSP9X cells, both in the absence and presence of cycloheximide (CHX) at a concentration of 10 μg/mL for a designated time (*n* = 3). **D** Western blot analysis was used to gauge the Beclin1 protein level in FLAG-tagged USP9X (WT or C417 mutant) HEK 293 cells, both with and without CHX at 10 μg/mL concentration for a specified duration (*n* = 3). **E** Lysates derived from GCs transfected with either Ad-shCtrl or Ad-shUSP9X underwent pretreatment with MG132 (*n* = 3), followed by subsequent immunoprecipitation and detection using the specified antibodies. **F** Lysates from Ad-Ctrl or Ad-USP9X transfected GCs were treated similarly to those in (**E**), followed by detection with specified antibodies after MG132 pretreatment (*n* = 3). **G** Lysates from HEK293T cells transfected with HA-tagged Ub, Myc-tagged Beclin1, and FLAG-tagged WT or C417A mutant USP9X underwent immunoprecipitation and subsequent anti-Myc, anti-HA, and anti-FLAG immunoblotting (*n* = 3). **H** In the presence or absence of TMX, GC cells were transfected with Ad-Ctrl or Ad-USP9X. Ub-Beclin1 was then detected by western blotting, utilizing anti-UB and anti-Beclin1 antibodies (*n* = 3). **I** HEK 293T cells were subjected to co-transfection with plasmids containing Myc-Beclin 1, FLAG-USP9X, and HA-Ub, designed explicitly for Lys0, Lys48, or Lys63 modifications, to assess the ubiquitination levels of Beclin1 (*n* = 3). **J** HEK 293T cells transfected with Ub WT or Ub Lys48R were cultured for 48 h in the presence of Ad-shCtrl or Ad-shUSP9X. Western blot analysis was then conducted to assess the protein levels of Beclin1 and USP9X (*n* = 3). Error bars represent the means ± SD. ^*^P < 0.05, ^**^P < 0.01

Further, we investigated the role of USP9X in modulating Beclin1 levels. Regardless of erastin or TMX treatment, USP9X knockdown resulted in a reduction in Beclin1 protein levels compared with the control treatment, whereas Beclin1 mRNA levels remained unaffected (Fig. S8A–F). This suggests that USP9X regulates the expression of Beclin1 protein through post-transcriptional modifications. Moreover, the depletion of USP9X led to a decline in Beclin1 protein level, which was reversed by the addition of the proteasome inhibitor MG132 (Fig. 7A). Then, we investigated the potential of USP9X to stabilize Beclin1 and observed that the overexpression of wild-type USP9X resulted in an increase in Beclin1 protein expression, whereas the overexpression of the catalytically inactive C471A mutant did not elicit such a response (Fig. 7B). Further, we investigated how USP9X depletion or mutation influences the stability of endogenous Beclin1 in the presence of cycloheximide, a protein synthesis inhibitor. The results demonstrated that Beclin1 exhibited a significantly shortened half-life in USP9X-deficient or mutant cells compared to control cells, indicating reduced protein stability (Fig. 7C and D). Moreover, USP9X knockdown markedly increased Beclin1 ubiquitination and decreased its protein abundance, while USP9X overexpression led to suppressed ubiquitination of Beclin1 and a corresponding increase in its protein levels (Fig. 7E and F). Then, we co-transfected HEK 293 T cells with vectors encoding Myc-Beclin1, FLAG-USP9X (WT or C471 mutant), and HA-Ub to investigate the impact of USP9X on Beclin1 ubiquitination. We found that the overexpression of wild-type USP9X significantly inhibited Beclin1 ubiquitination, whereas the C471A mutant USP9X counteracted this effect (*P* < 0.01; Fig. 7G). Furthermore, USP9X exerted an additional inhibitory effect on the ubiquitination of Beclin1 and enhanced its stability in the presence of TMX (Fig. 7H). These results indicate that USP9X directly stabilizes Beclin1 by deubiquitinating it. Next, we investigated the type of Beclin1 polyubiquitination affected by USP9X and observed that Lys48-linked Beclin1 polyubiquitination was efficiently decomposed by USP9X, whereas non-degradable Lys63 remained unaffected (Fig. 7I). To investigate whether USP9X stabilizes Beclin1 expression through the polyubiquitination of Lys-48 links, we introduced Lys48-resistant (Lys48R) ubiquitin into cells with silenced USP9X. The results revealed that the enforced expression of Lys48R ubiquitin mitigated the downregulation of Beclin1 caused by USP9X silencing, indicating the critical role of Lys48-linked polyubiquitination in USP9X-mediated Beclin1 turnover (Fig. 7J). Collectively, these findings suggested that USP9X functions as a bona fide deubiquitinase targeting Beclin1 for deubiquitination.

### USP9X regulates ferroptosis through Beclin1

**Fig. 8:**
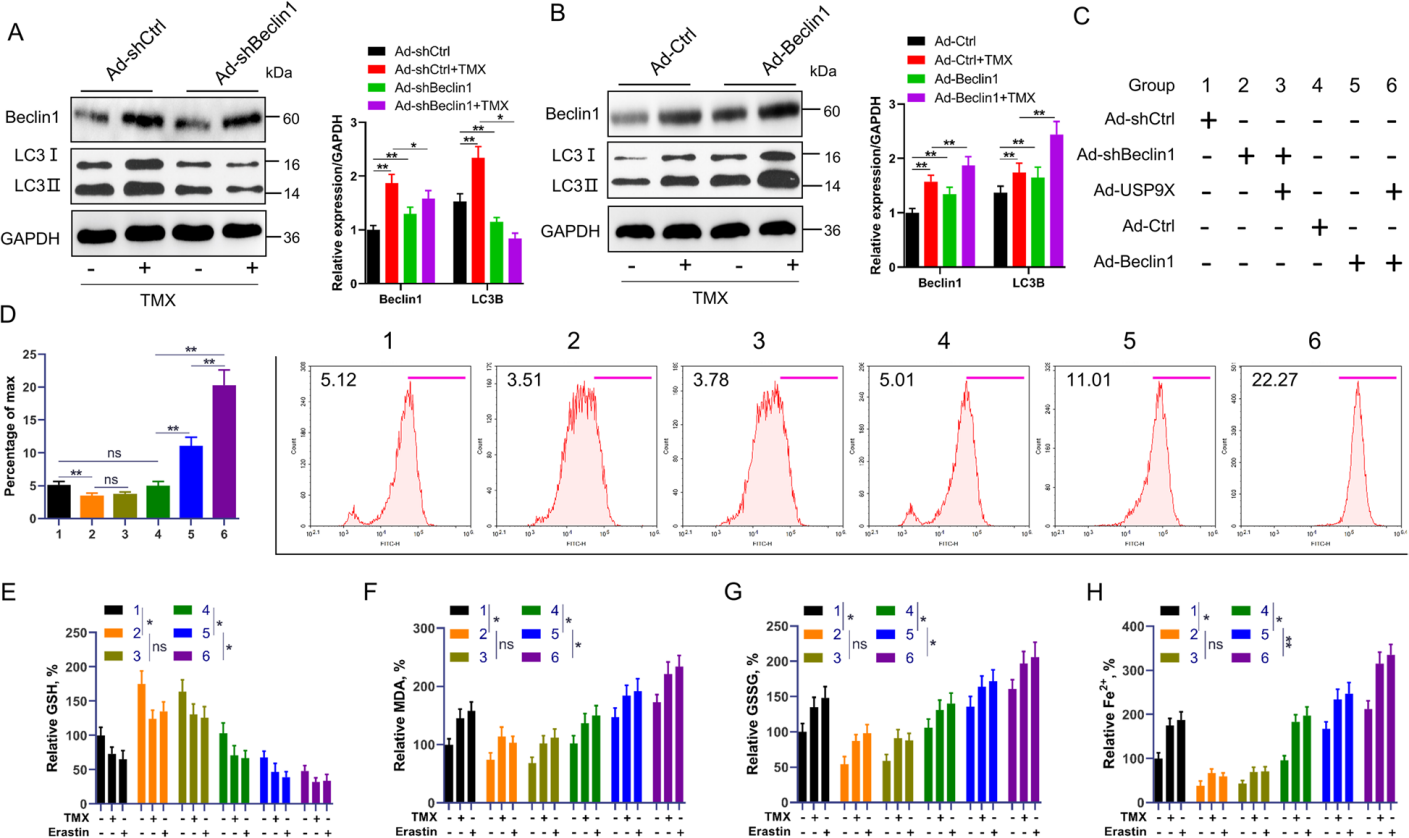
Modulation of ferroptosis signaling via USP9X regulation of Beclin1 in GCs. GCs underwent transfection with either Ad-shBeclin1 or Ad-Beclin1 constructs, followed by subsequent treatment with erastin (5 µmol/L) for 6 h or with TMX for 12 h. **A** Western blot analysis assessed the expression levels of Beclin1 and LC3B proteins in control cells and cells with silenced Beclin1 (*n* = 3). GAPDH served as the loading control. **B** The expression levels of Beclin1 and LC3B proteins in control cells and cells with overexpressed Beclin1 were determined using western blot analysis (*n* = 3). GAPDH was employed as a loading control. **C** Comprehensive details regarding the experimental group in this specific experiment phase are provided herein. **D** The lipid peroxidation levels in the indicated cells were evaluated through flow cytometry (*n* = 6). **E**–**H** The indicated cells were treated with erastin or TMX, and the intracellular levels of GSH, MDA, iron, and GSSG were measured (*n* = 6). Error bars represent the means ± SD. ^*^P < 0.05, ^**^P < 0.01. Non-significant differences were denoted with the notation "ns"

To determine whether USP9X regulates ferroptosis through Beclin1-mediated autophagy, we employed Beclin1 expression using adenoviral vectors: Ad-shBeclin1 to suppress autophagy and Ad-Beclin1 to enhance it. Western blot analysis confirmed the efficiency of these interventions transfection with Ad-shBeclin1 led to a significant reduction in Beclin1 and LC3B protein levels, whereas Ad-Beclin1 transfection significantly elevated the expression of both proteins (*P* < 0.01; Fig. 8A and B). Figure 8C presents detailed information about the experimental test groups. Subsequently, we investigated various prototypical ferroptosis events in GCs co-treated with Ad-shBeclin1 and Ad-Beclin1. Pretreatment with Ad-shBeclin1 eliminated ferroptosis events irrespective of Ad-USP9X treatment. Conversely, compared with Ad-Beclin1 treatment alone, co-treatment of Ad-Beclin1 and Ad-USP9X results in an upregulation of ferroptosis events (Fig. 8D–H). In addition, western blotting confirmed that Ad-shBeclin1 preconditioning decreased ACSL4 and increased GPX4 and FTH1 levels irrespective of whether USP9X was silenced or overexpressed (Fig. S9A). In addition, the knockdown of Beclin1 induced resistance against the inhibitory effects of erastin or TMX on cell viability, whereas the upregulation of Beclin1 significantly increased erastin or TMX-induced cell death (*P* < 0.05; Fig. S9B and C). Interestingly, PI staining revealed that USP9X overexpression significantly enhanced the reduction in cell viability when combined with Beclin1 overexpression, indicating a synergistic effect. In contrast, USP9X overexpression had no discernible impact on cell viability in cells where Beclin1 was silenced (Fig. S9D). These results suggest that USP9X promotes ferroptosis primarily through the modulation of Beclin1-dependent autophagy.

## Discussion

Follicular atresia is a fundamental physiological process that occurs throughout the development, maturation, and ovulation of ovarian follicles [32]. It is widely accepted that the death of GCs is the principal driver of follicular atresia, with programmed cell death pathways including autophagy, apoptosis, and necroptosis playing central roles [33, 34]. However, the mechanisms underlying GC death during atresia remain incompletely understood. Ferroptosis, a recently identified form of programmed cell death, has received limited attention in female reproduction. Given their distinctive follicular architecture and well-defined developmental stages, poultry represent an ideal model for studying ovarian disorders [35]. To our knowledge, the present study is the first to demonstrate that ferroptosis contributes to the pathogenesis of follicular atresia in birds. We showed in this present study that activation of ferroptosis promotes follicular atresia, whereas its pharmacological inhibition mitigates TMX-induced atresia, suggesting a therapeutic potential for ferroptosis suppression. Additionally, through both gain- and loss-of-function experiments, we identified USP9X as a key regulator of ferroptosis in avian GCs. USP9X activates autophagy by interacting with Beclin1, leading to ferritin degradation, iron accumulation, and subsequent ferroptosis. These findings suggest that USP9X may serve as a promising therapeutic target for conditions associated with aberrant follicular atresia.

Increasing evidence suggests that USP9X plays a crucial role in the regulation of female reproductive function. Fassnacht et al. [36] discovered that the loss or mutation of *USP9X* results in premature ovarian failure in women. Moreover, Sato et al. [37] reported a high expression of USP9X in GCs but low expression in membrane and stromal cells. This implies that USP9X may exert a crucial influence on folliculogenesis by modulating the growth and development of GCs. Recently, USP9X has been found to exhibit targeted regulatory interactions with several ferroptosis regulators. Huang and Zhao [27] found that USP9X activates the antioxidant response element and alleviates the pathological process of diabetic renal fibrosis by deubiquitinating Nrf2. In another study by Chen et al. [38], USP9X was found to regulate the proliferation of hepatoma cells by stabilizing the protein level of β-catenin, which is known to promote GPX4 transcription by binding to the TCF4, thereby controlling the ferroptosis pathway [39]. Thus, USP9X may have a significant effect on iron metabolism or homeostasis by modulating the proteins involved in ferroptosis pathways. In the current study, we observed a significant upregulation of USP9X in the GCs of atretic follicles, further validating its role in ferroptosis regulation. GC death is a dynamic process involving multiple, often interconnected, cell death pathways. Thus, USP9X may influence follicular atresia through the coordinated modulation of ferroptosis and other death mechanisms.

Several studies have highlighted the close interplay between ferroptosis and autophagy [40]. Autophagy, particularly ferritinophagy, acts upstream of ferroptosis by facilitating intracellular iron release and reactive oxygen species (ROS) accumulation [41]. Consistent with these findings, our data indicate that USP9X enhances autophagy, likely contributing to its pro-ferroptotic effect. We further demonstrated that USP9X induces autophagy by stabilizing Beclin1, a core autophagy regulator. Although mTOR is a well-known modulator of autophagy [42], our results showed no significant changes in mTOR or AMPK protein levels upon USP9X overexpression. This suggests that USP9X promotes autophagy primarily via Beclin1 stabilization rather than through classical AMPK/mTOR signaling. However, we observed no impact of USP9X on the levels of AMPK and mTOR proteins in GCs; instead, USP9X was found to directly induce autophagy by stabilizing Beclin1.

Beclin1 plays a central role in autophagy by recruiting autophagy-related proteins to the phagophore, thus facilitating autophagosome formation and maturation [43]. Beyond autophagy, Beclin1 has also been implicated in ferroptosis [44]. Song et al. [45] reported that Beclin1 directly inhibits system Xc^−^ by interacting with the core protein involved in ferroptosis. Beclin1 also interacts with p53, promoting its degradation and consequently suppressing SLC7A11, a key anti-ferroptotic gene [46]. Furthermore, Beclin1-mediated autophagy promotes ferritin degradation, leading to increased intracellular iron and ferroptosis induction [47]. These studies indicate that Beclin1 can facilitate the degradation of ferritin and trigger ferroptosis through the autophagy pathway. In addition, Beclin1 positively regulates system Xc^−^ inhibitor-induced ferroptosis in an autophagy-independent manner [48]. In our study, inhibition of autophagy using CQ or ATG7 knockdown partially suppressed USP9X-induced ferroptosis, supporting the dual role of Beclin1 in both autophagy-dependent and independent ferroptosis. Given its critical role and regulation by the ubiquitin–proteasome system, Beclin1 has emerged as a promising target for therapeutic modulation in various diseases [49]. Therefore, it is crucial to identify the regulatory mechanisms governing Beclin1 expression to develop targeted therapies for the disease. The present study determined that USP9X is a bona fide de-ubiquitinase that interacts with Beclin1 to reverse its polyubiquitination and protect it from proteasomal degradation, thereby activating Beclin1-mediated ferroptosis. In addition, silencing Beclin1 reversed the ferroptotic effects induced by USP9X overexpression, confirming that Beclin1 stability is essential for USP9X's function in regulating GCs ferroptosis. While our findings highlight a conserved USP9X-Beclin1-ferroptosis axis in avian follicular atresia, comparative analysis suggest both shared and species-specific regulatory mechanisms. In mammals, for example, USP9X regulates autophagy through alternative substrates such as Raptor, whose deubiquitination impairs autophagy in neurodegenerative models (P301S mice) [50]. In contrast, our results show that in avian GCs, USP9X stabilizes Beclin1 to promote autophagy and ferroptosis. These functional differences may reflect unique reproductive traits in birds, such as hierarchical follicular development and rapid ovulatory cycles, which impose distinct metabolic and regulatory demands. The sequential recruitment and atresia of preovulatory follicles in poultry likely sensitize GCs to ferroptosis, particularly in the context of disrupted iron homeostasis. This study establishes the pivotal role of USP9X in inducing GC ferroptosis via Beclin1 stabilization, as well as opens new avenues for future studies. Given the involvement of USP9X in multiple cell death pathways, its broader regulatory network in follicular atresia potentially involving crosstalk with apoptosis, necroptosis, and autophagy warrants further investigation. Understanding these interactions may ultimately provide novel strategies for managing ovarian dysfunction and enhancing reproductive performance.

## Conclusions

**Fig. 9:**
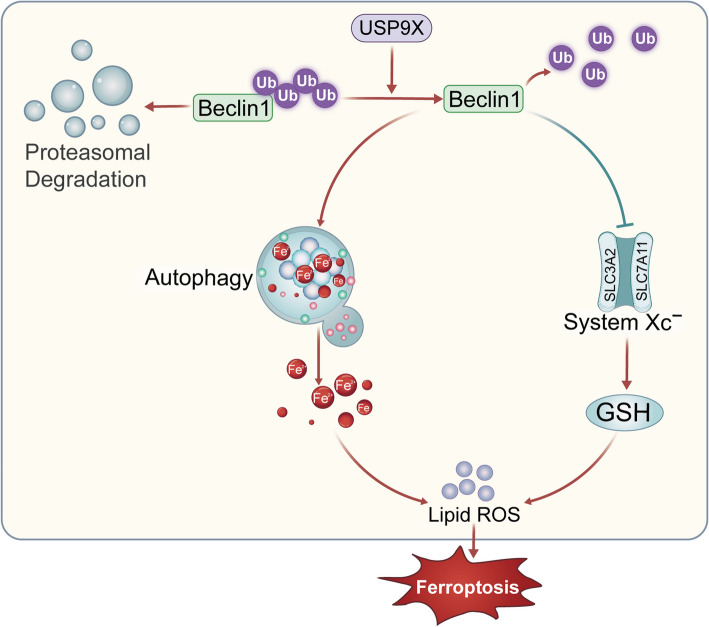
Schematic representation of USP9X's role in follicular atresia. This diagram visually represents how USP9X stabilizes Beclin1 expression by deubiquitinating it. This stabilization activates autophagy, promoting ferritin degradation and lipid peroxidation, ultimately inducing ferroptosis

This study identifies ferroptosis as a key contributor to follicular atresia in a TMX-induced avian model, mediated by the USP9X-Beclin1 signaling axis. Specifically, USP9X promotes ferroptosis in granulosa cells by stabilizing Beclin1 via de-ubiquitination, thereby activating autophagy-dependent ferritin degradation and iron accumulation (Fig. 9). These findings offer valuable insights into the molecular mechanisms underlying follicular degeneration and highlight USP9X and Beclin1 as potential genetic markers for enhancing follicular development and reproductive efficiency in poultry breeding programs.

## Supplementary Information

Additional file 1: Table S1 The primers used in this study.

Additional file 2: Fig. S1 Impact of TMX treatment on gene expression in bird follicles. Fig. S2 Iron overload induces follicular atresia. Fig. S3 Induction of ferroptosis in GCs by erastin, RSL3, and sorafenib. Fig. S4 Effects of liproxstatin-1 on ferroptosis and functional recovery in bird follicles following TMX treatment. Fig. S5 Erastin's regulation of cell death through the ferroptosis pathway in GCs. Fig. S6 USP9X overexpression's effects on ferroptosis and TMX-induced follicular atresia. Fig. S7 Rescue of USP9X-induced ferroptosis through inhibition of the autophagy signaling pathway. Fig. S8 USP9X's modulation of Beclin1 protein expression without affecting mRNA levels. Fig. S9 The effect of USP9X-regulated Beclin1 on GCs viability.

## Data Availability

RNA-seq data used in this investigation have been submitted to the China National Center Data Center for Bioinformation under accession code CRA010052. All data underlying the results are available in the main article or in the supplementary data files.

## Abbreviations

ACSL4: Acyl-CoA synthetase long chain family member 4
CCK8: Cell Counting Kit-8
CQ: Chloroquine
FTH1: Ferritin heavy chain 1
GC: Granulosa cell
GPX4: Glutathione peroxidase 4
GSH: Glutathione
LC3B: MAP1LC3B
Lip-1: Liproxstatin-1
PCD: Programmed cell death
TMX: Tamoxifen
USP9X: Ubiquitin-specific peptidase 9, X-linked
