# Supplementary material for: USP9X-triggered ferroptosis mediates follicular atresia via deubiquitinating Beclin1 in chicken
Source: J Anim Sci Biotechnol. 2025 Oct 23;16:134. doi: 10.1186/s40104-025-01269-8 (PMC12548185; doi:10.1186/s40104-025-01269-8)
Supplement: Supplementary file 2 — Additional file 2: Fig. S1 Impact of TMX treatment on gene expression in bird follicles. Fig. S2 Iron overload induces follicular atresia. Fig. S3 Induction of ferroptosis in GCs by erastin, RSL3, and sorafenib. Fig. S4 Effects of liproxstatin-1 on ferroptosis and functional recovery in bird follicles following TMX treatment. Fig. S5 Erastin's regulation of cell death through the ferroptosis pathway in GCs. Fig. S6 USP9X overexpression's effects on ferroptosis and TMX-induced follicular atresia. Fig. S7 Rescue of USP9X-induced ferroptosis through inhibition of the autophagy signaling pathway. Fig. S8 USP9X's modulation of Beclin1 protein expression without affecting mRNA levels. Fig. S9 The effect of USP9X-regulated Beclin1 on GCs viability. [file 40104_2025_1269_MOESM2_ESM.docx]

**Additional file 2**


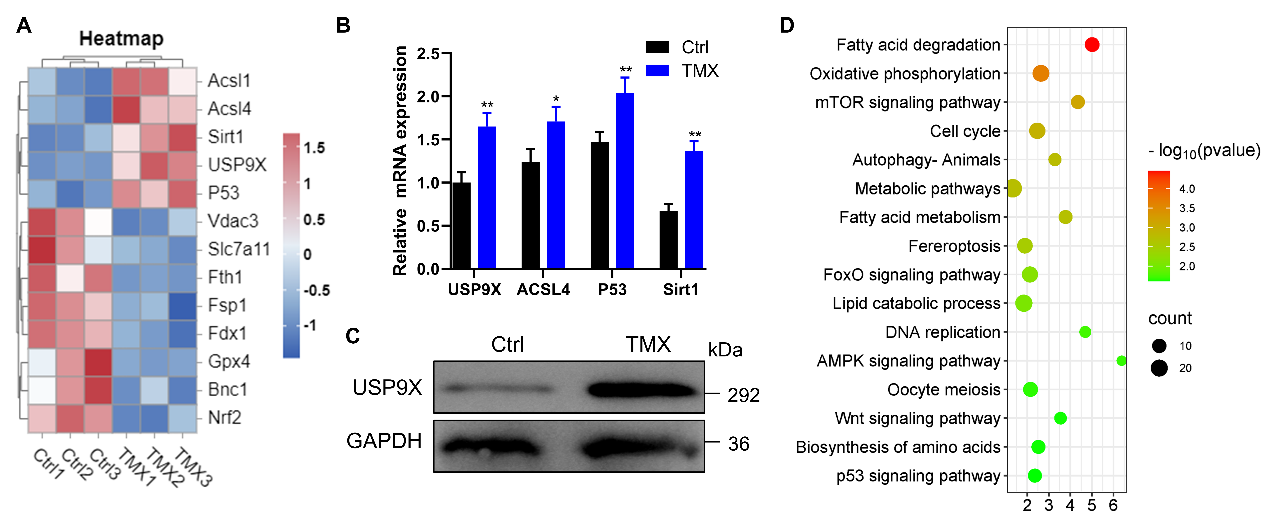


**Fig. S1** Impact of TMX treatment on gene expression in bird follicles. **A** Hierarchical clustering and heatmap showing significant differentially expressed genes in bird follicles following 9-day treatment with TMX or a vehicle (*n* = 3). **B** Graphical representation of the expression of ferroptosis target genes in bird follicles treated with TMX or a vehicle for 9 days (*n* = 3). **C** Western blot analysis depicting the levels of USP9X protein in bird follicles after a 9 d treatment with TMX or a vehicle (*n* = 3). **D** A diagram showing the pathway enrichment analysis of significantly differentially expressed genes between follicles treated with TMX or a vehicle in birds (*n* = 3). Error bars represent the means ± SD. ^*^*P* < 0.05, ^**^*P* < 0.01. Student’s *t*-test


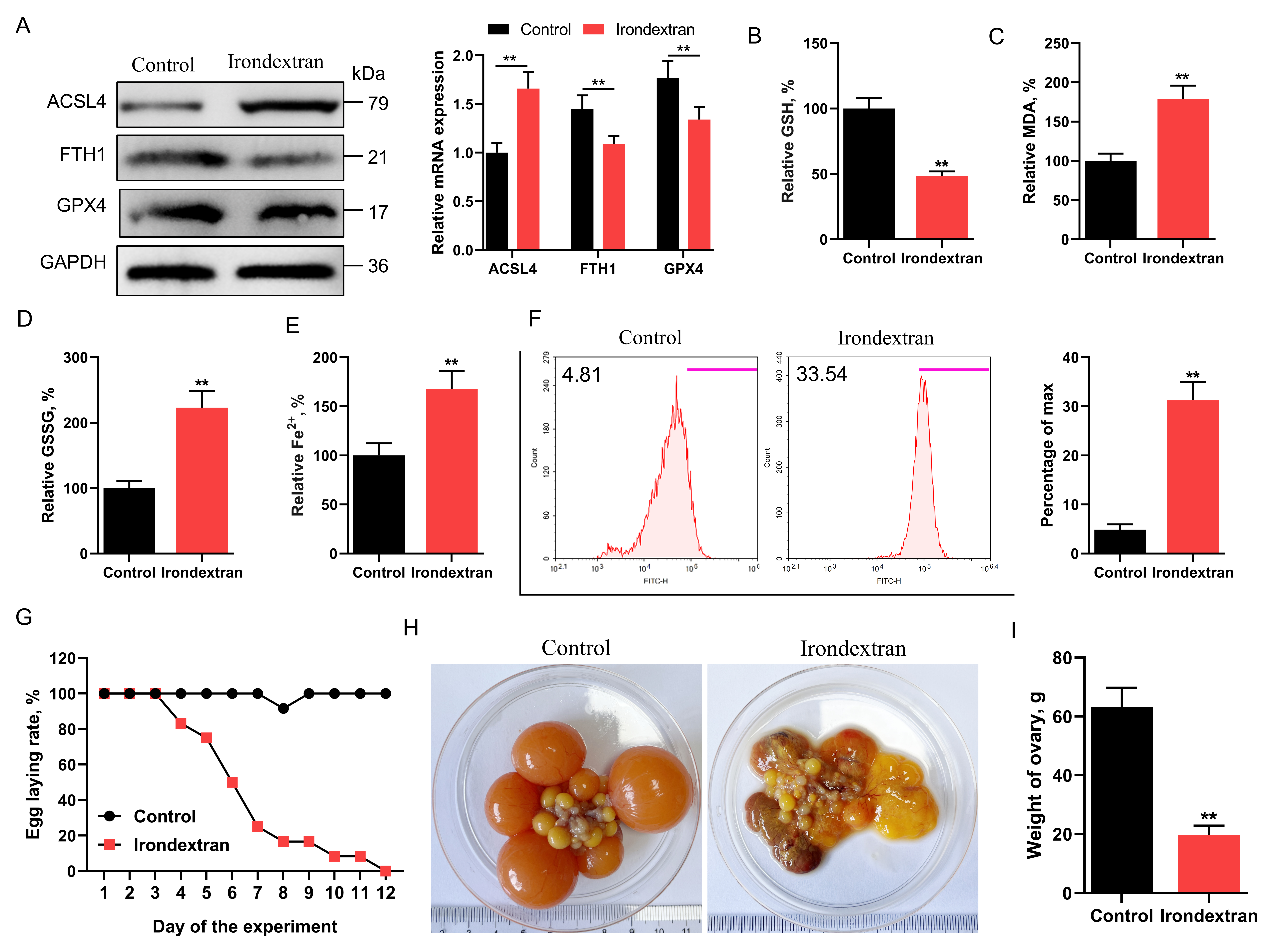


**Fig. S2** Iron overload induces follicular atresia. **A** Western blot evaluation of ACSL4, FTH1, and GPX4 protein levels in bird follicles during Irondextran treatment (*n* = 12). **B-F** Graphs illustrating the relative concentrations of GSH, MDA, iron content, and GSSG in bird follicles throughout Irondextran treatment (*n* = 12). **G-I** Assessment of egg-laying rate, ovary morphology, and ovary weights in birds (*n* = 12) following treatment with Irondextran or vehicle (PBS; control). Error bars represent the means ± SD. ^*^*P* < 0.05, ^**^*P* < 0.01. Student’s *t*-test

**
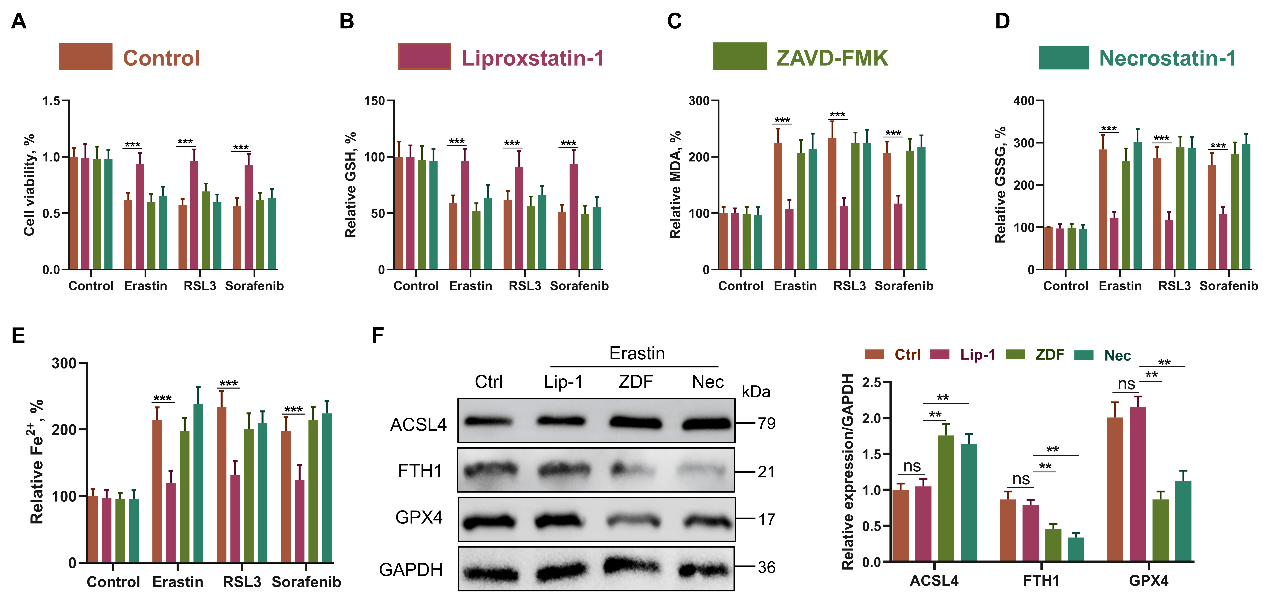
**

**Fig. S3** Induction of ferroptosis in GCs by Erastin, RSL3, and Sorafenib. GCs were treated with erastin (10 μmol/L), sorafenib (10 μmol/L), or RSL3 (2.5 μmol/L), with or without inhibitors, for 24 hours. (A) Cell viability measured by the CCK8 assay (*n* = 6). (B-E) Graphs show MDA production, iron accumulation, GSH, and GSSG levels under different intervention conditions (*n* = 6). (F) Western blot analysis displaying ACSL4, FTH1, and GPX4 protein expression levels under varying conditions (*n* = 3). Error bars represent the means ± SD. ^*^*P* < 0.05, ^**^*P* < 0.01. Student’s *t*-test. Non-significant differences were denoted with the notation "ns"


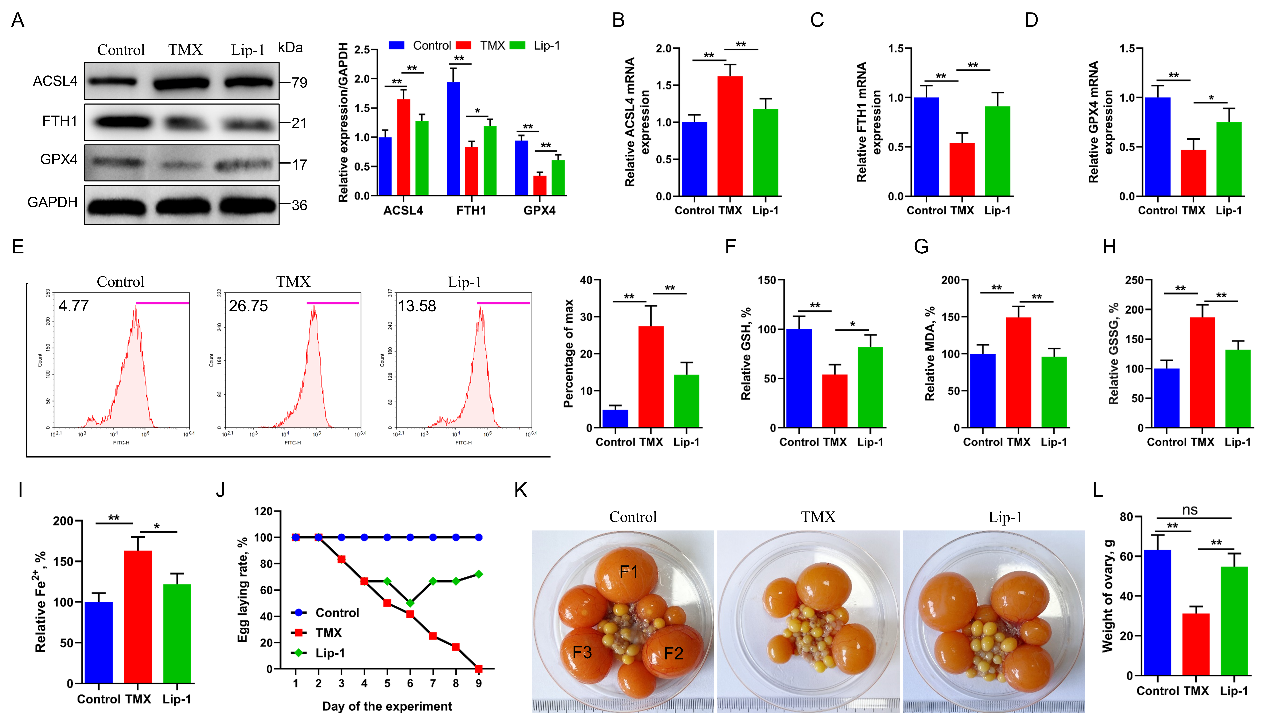


**Fig. S4** Effect of liproxstatin-1 on ferroptosis and functional recovery in bird follicles following TMX treatment. The birds were intraperitoneally administered TMX, TMX + Liproxstatin-1, or ethanol (control) for a duration of 9 d, TMX (6 mg/kg), Liproxstatin-1 (5 mg/kg). **A** After applying TMX or Liproxstatin-1 (Lip-1, 6 mg/kg), Western blot analysis assessed the protein levels correlated with ferroptosis in avian follicles (*n* = 3). **B-D** qPCR analysis was employed to evaluate the expression levels of proteins associated with ferroptosis in avian follicles after treatment with either TMX or Lip-1 (*n* = 3). **E-I** Measurements of the relative values of lipid peroxidation, GSH, MDA, Iron, and GSSG concentrations in bird follicles after treatment with TMX or Lip-1 were taken (*n* = 6). **J-L** The egg-laying rate, ovarian morphology, and ovarian weight were assessed following treatment with TMX or Lip-1 (*n* = 12). Error bars represent the means ± SD. ^*^*P* < 0.05, ^**^*P* < 0.01. Student’s *t*-test

**
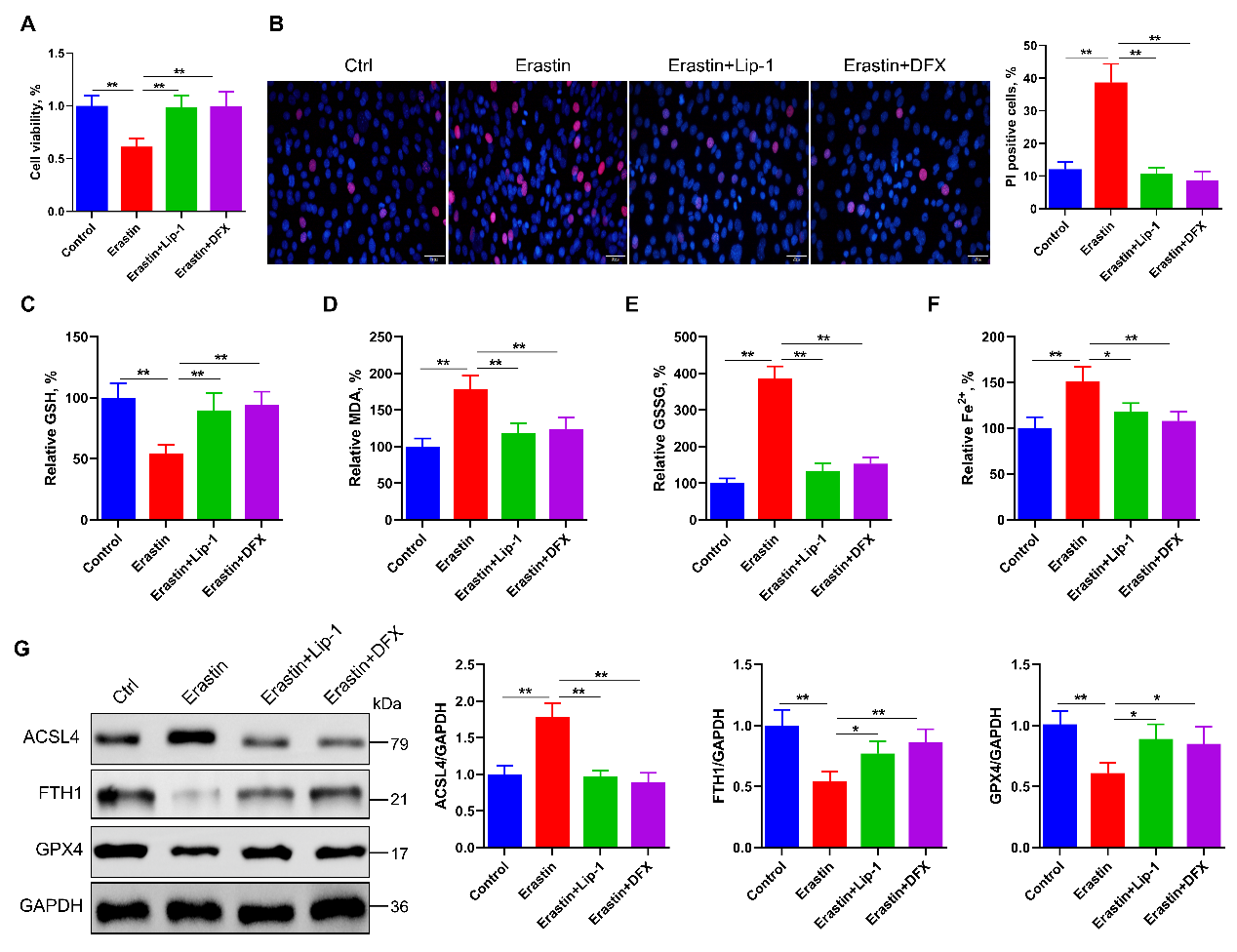
**

**Fig. S5** Erastin's regulation of cell death through the ferroptosis pathway in GCs. GCs were treated with erastin (5 μmol/L) for 12 hours, with or without Liproxstatin-1 (100 nmol/L) or Deferasirox (50 μmol/L). **A** The CCK8 assay detected cell viability (*n* = 6). **B** PI staining was employed to assess cell viability under the same conditions (*n* = 6). **C-F** Graphs showing GSH, MDA, 4-HNE, and iron content measurements under different conditions (*n* = 6). **G** Western blot analysis was employed to quantitate the protein expression levels of ACSL4, FTH1, and GPX (*n* = 3). Error bars represent the means ± SD. ^*^*P* < 0.05, ^**^*P* < 0.01. Student’s *t*-test


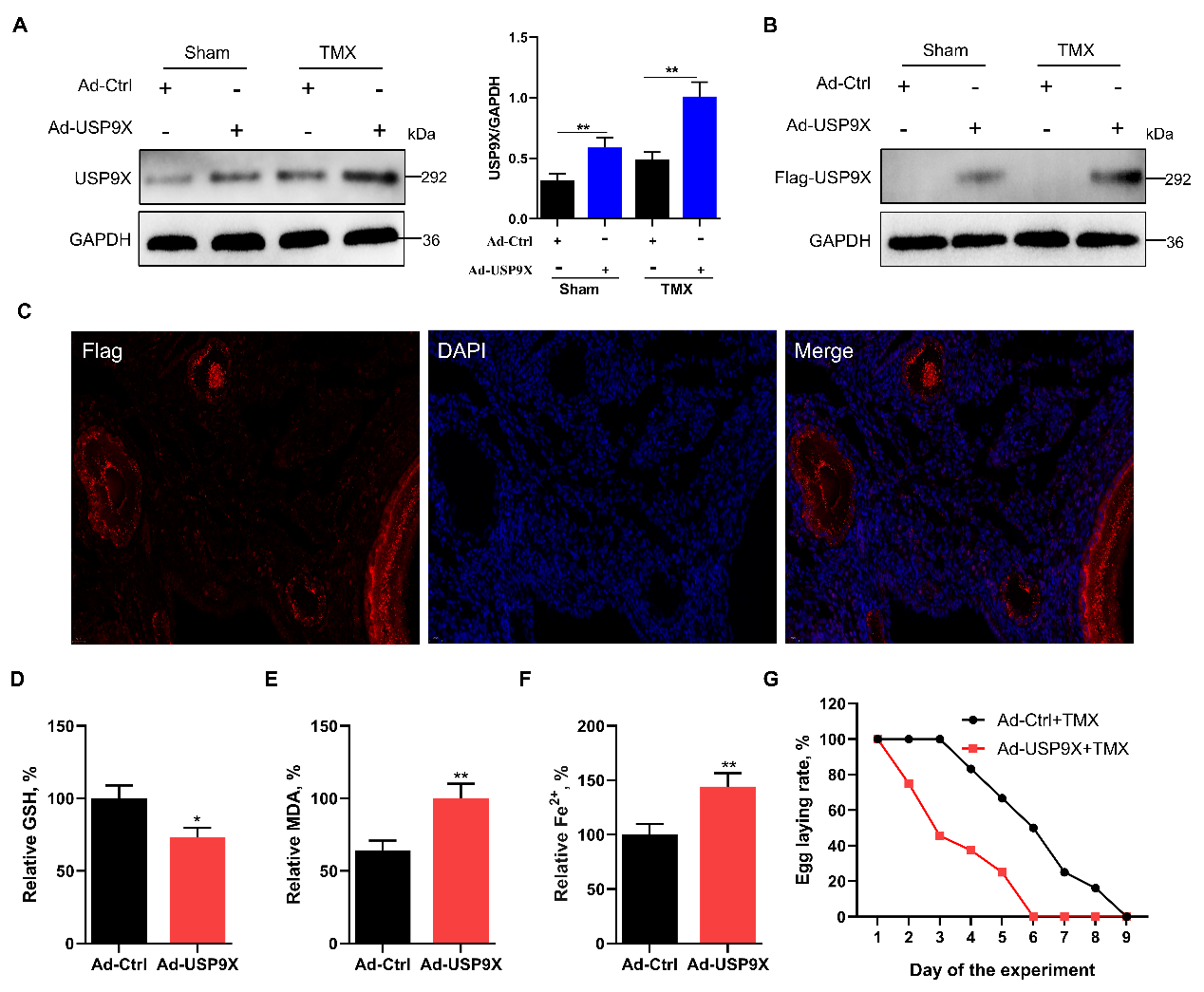


**Fig. S6** USP9X overexpression's effects on ferroptosis and TMX-induced follicular atresia. **A** and **B** Western blot analysis to assess USP9X transfection efficiency in cells treated with or without TMX (*n* = 3). **C** Representative immunofluorescence images for identifying Ad-USP9X transfection in follicle (*n* = 3). **D-F** Graphs displaying the GSH, MDA, and Iron concentrations in bird follicles transfected with either Ad-Ctrl or Ad-USP9X (*n* = 6). **G** A diagram illustrating the egg-laying rate in birds transfected with Ad-USP9X or Ad-Ctrl and treated with TMX. Error bars represent the means ± SD. ^*^*P* < 0.05, ^**^*P* < 0.01. Student’s *t*-test


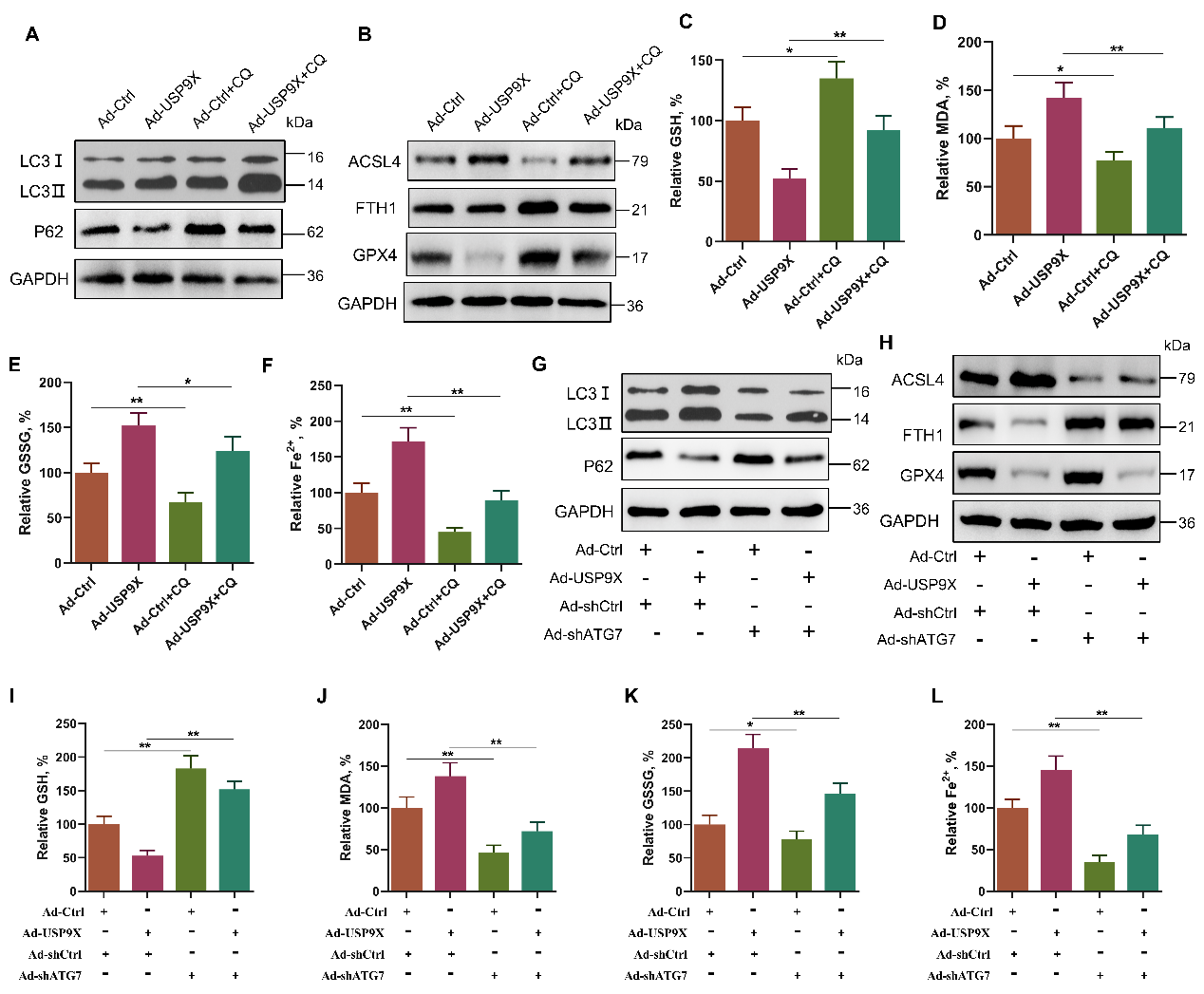


**Fig. S7** Rescue of USP9X-induced ferroptosis through inhibition of the autophagy signaling pathway. GCs were treated with either CQ at a concentration of 20 μmol/L or were transfected with Ad-shATG7 for 24 hours. **A** Western blot analysis was performed to quantify the expression levels of LC3B and P62 proteins following transfection with either USP9X or control in the presence or absence of CQ treatment (*n* = 3). **B** Western blot analysis evaluated the expression levels of proteins associated with ferroptosis after transfection with either USP9X or control, under conditions with or without CQ treatment (*n* = 3). **C-F** The GSH, MDA, GSSG, and Iron concentrations were measured after transfection with USP9X or control, with or without CQ treatment (*n* = 6). **G** Western blot analysis was conducted to ascertain the expression levels of LC3B and P62 proteins subsequent to co-transfection with Ad-USP9X, Ad-sh-ATG7, or Ad-Ctrl constructs (*n* = 3). **H** Western blot analysis was performed to evaluate the expression levels of ferroptosis-associated proteins ACSL4, FTH1 and GPX4 after co-transfection with Ad-USP9X, Ad-sh-ATG7, or Ad-Ctrl cells (*n* = 3). **I-L** The GSH, MDA, GSSG, and Iron concentrations were measured after co-transfection with Ad-USP9X, Ad-sh-ATG7, or Ad-Ctrl cells (*n* = 6). Error bars represent the means ± SD. ^*^*P* < 0.05, ^**^*P* < 0.01. Student’s *t*-test


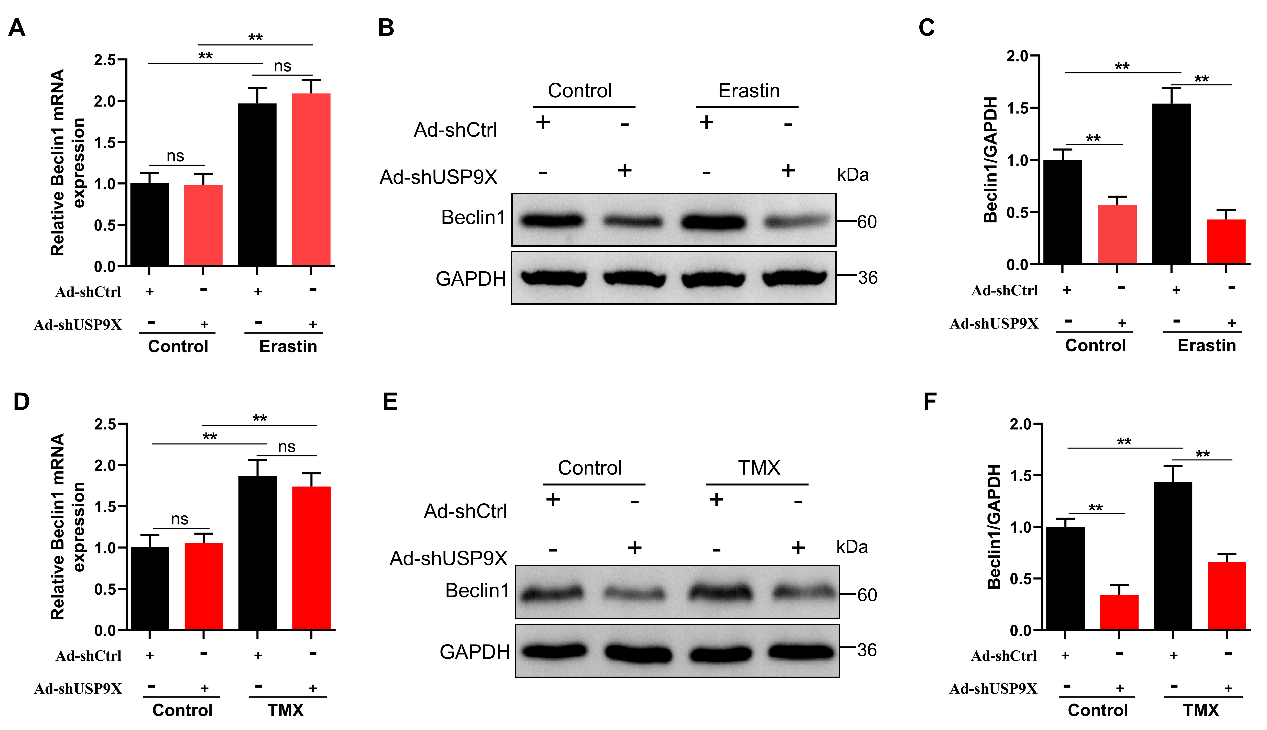


**Fig. S8** USP9X's modulation of Beclin1 protein expression without affecting mRNA levels. **A** qPCR analysis of Beclin1 mRNA expression in Ad-shCtrl or Ad-USP9X cells after treatment with Erastin (5 µmol/L, 6 h) (*n* = 3). **B** and **C** Western blot analysis showing Beclin1 protein expression in Ad-shCtrl or Ad-USP9X cells after treatment with Erastin (*n* = 3). **D** qPCR analysis of Beclin1 mRNA expression in Ad-shCtrl or Ad-USP9X cells after treatment with TMX (2 µmol/L, 12 h) (*n* = 3). **E** and **F** Western blot analysis depicting Beclin1 protein expression in Ad-shCtrl or Ad-USP9X cells following treatment with TMX (*n* = 3). Error bars represent the means ± SD. ^*^*P* < 0.05, ^**^*P* < 0.01. Student’s *t*-test. Non-significant differences were denoted with the notation "ns"

**
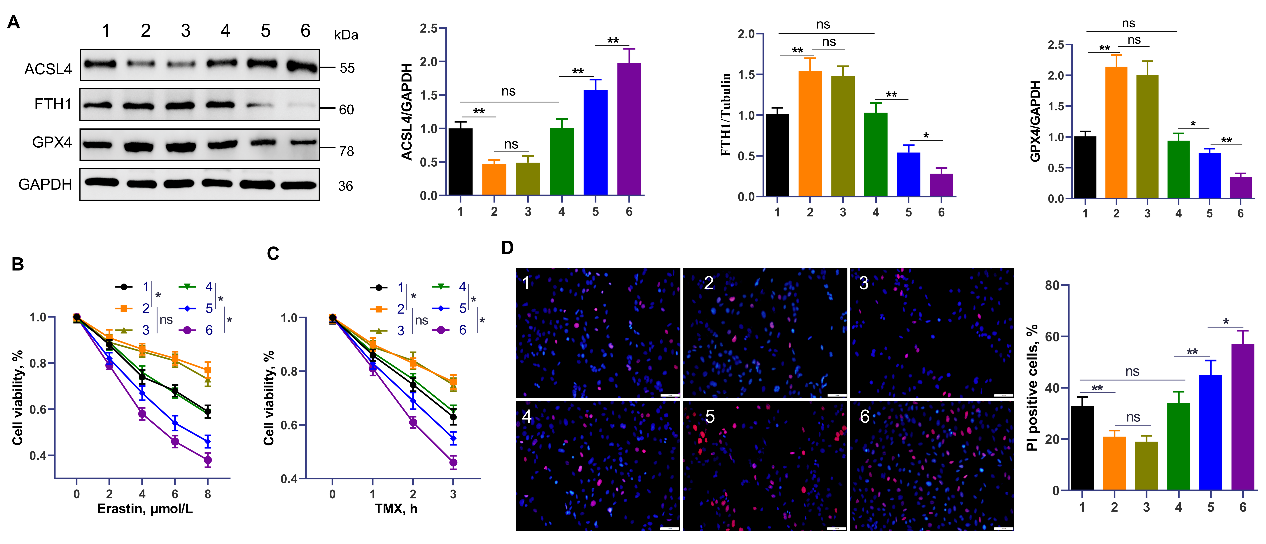
**

**Fig. S9** The effect of USP9X-regulated Beclin1 on GCs viability. **A** We performed Western blot analysis to detect the ACSL4, FTH1, and GPX4 expression in the indicated cells (*n* = 3). GAPDH functioned as a loading control. **B** and **C** Cell viability was examined using the CCK-8 assay under Erastin or TMX treatment conditions (*n* = 6). **D** Cell survival was evaluated by PI staining in the indicated cells (*n* = 3). Error bars represent the means ± SD. ^*^*P* < 0.05, ^**^*P* < 0.01. Student’s *t*-test. Non-significant differences were denoted with the notation "ns"
